# Kupffer cells are key mediators of antigen-specific immune tolerance by peptide-coupled red blood cells

**DOI:** 10.1126/sciadv.aeg6770

**Published:** 2026-10-07

**Authors:** Vasileia Kalaitzaki, Pietro Oldrati, Filipa M. Ferreira, Antonios Katsoulas, Ahmed Ali, Livio Baselgia, Yun Liu, Jincheng Fang, Roman Sankowski, Mai Matsushita, Florence Vallelian, Alexandre P. A. Theocharides, Cyrill Géraud, Roland Martin, Andreas Lutterotti, Thorsten Buch

**Affiliations:** ^1^ Institute of Laboratory Animal Science, University of Zurich, Schlieren, Switzerland.; ^2^ Cellerys AG, Wagistrasse 21, Schlieren, Switzerland.; ^3^ Department of Internal Medicine, University Hospital and University of Zurich, Zurich, Switzerland.; ^4^ Institute for Neuropathology, University Hospital Freiburg, Freiburg, Germany.; ^5^ Department of Medical Oncology and Hematology, University of Zurich and University Hospital, Zurich, Switzerland.; ^6^ Section of Molecular and Clinical Dermatology, Department of Dermatology, Venereology and Allergology and European Center for Angioscience, University Medical Center and Medical Faculty Mannheim, Heidelberg University, Mannheim, Germany.; ^7^ Institute of Experimental Immunology, University of Zurich, Zurich, Switzerland.; ^8^ Department of Clinical Neurosciences, Karolinska Institute, Stockholm, Sweden.

## Abstract

Induction of antigen-specific immune tolerance is the most precise method for reverting an autoreactive immune response in diseases such as multiple sclerosis (MS). Coupling red blood cells (RBCs) with a cocktail of MS immunodominant peptides is a promising approach to induce antigen-specific immune tolerance in patients. Here, we show that RBCs coupled with disease-inducing antigen efficiently prevent the development of experimental autoimmune encephalomyelitis, when administered prophylactically, and ameliorate disease symptoms when given therapeutically. The tolerogenic activity of peptide-coupled RBCs (pcRBC) was antigen specific and dependent on both cell and peptide dose. Kupffer cells (KC) were the primary phagocytes of pcRBC, and their uptake of pcRBC did not require complement component 3, SIRPα, and Stabilin 1 and 2. KC were indispensable for the induction of tolerance, which was accompanied by programmed death-ligand 1 up-regulation and a tolerogenic transcriptional shift. Our findings establish KC as central mediators of pcRBC-induced immune tolerance and provide mechanistic insight for the use of this therapeutic strategy in autoimmune disease.

## INTRODUCTION

Multiple Sclerosis (MS) is a chronic autoimmune disease of the central nervous system (CNS), which predominantly manifests in young adults and affects women three times more often than men (*1*). MS pathology is characterized by CD4^+^ T cells, B cells, and myeloid cell infiltration into the CNS and induction of a local inflammatory response, which results in demyelination, axonal or neuronal damage, glial cell activation, and metabolic changes (*1*). Key drivers of the immunopathogenesis are autoreactive high avidity T helper 1 (T_H_1) or T_H_1* cells, which target both myelin- and nonmyelin antigens, such as myelin basic protein, myelin oligodendrocyte glycoprotein (MOG), proteolipid protein (*2*), RAS guanyl-releasing protein 2, guanosine diphosphate–fucose synthase (*3*, *4*), and additionally the recently found autoantigens (*5*). Furthermore, CNS-resident cells, particularly activated astrocytes and microglia, are critical effectors of the disease response (*6*, *7*).

Approved disease-modifying therapies for MS are highly effective in reducing disease activity but cause nonspecific immunomodulation or suppression, which is associated with long-term safety risks (*8*, *9*). Consequently, efforts are intensifying toward developing antigen-specific therapies for restoring immune tolerance without compromising protective immune functions (*10*, *11*).

Among the strategies to induce antigen-specific tolerance that have been explored, Miller *et al.* (*12*) pioneered the intravenous injection of antigen-coupled splenocytes (pcSplenocytes) or peptide-coupled red blood cells (pcRBC)—through cross-linking with either cell lysate, proteins, or peptides using 1-ethyl-3-(3-dimethylaminopropyl)carbodiimide hydrochloride (EDC). In several models of T cell–mediated autoimmune diseases, this strategy showed excellent efficacy including in relapsing-remitting experimental autoimmune encephalomyelitis (RR-EAE) in mice (*13*–*18*) and rats (*19*), Theiler’s virus–mediated demyelination (*20*), type 1 diabetes (*21*), allogeneic islet cell transplantation (*22*), and an allergy model (*23*). In EAE, this protocol was described as exquisitely antigen specific and highly efficacious in preventing and treating ongoing disease. Mechanistically, the coupling process induces apoptosis of the coupled cells, which leads to their phagocytosis by splenic marginal zone macrophages after intravenous injection. It has been suggested that these antigen-presenting cells (APCs) process and present the coupled antigens, leading to indirect immune tolerance (*17*, *18*). They also expressed interleukin-10 (IL-10) and programmed death-ligand 1 (PD-L1), and T regulatory cells were shown to be required for long-term maintenance of the induced tolerance (*18*).

Building on this foundation, we undertook a phase Ib clinical trial in patients with MS and demonstrated the safety and tolerability of autologous peripheral blood mononuclear cells (PBMCs) coupled with seven myelin peptides. We provided evidence for the induction of immune tolerance in patients receiving the highest dose of PBMCs (*24*). To overcome the limitations in the collection and storage of PBMCs and to target the liver as a tolerogenic organ (*25*), we continued the development of the approach using RBCs as tolerogenic carrier cells. Our recent phase Ib/IIa clinical trial in patients with MS demonstrated the safety and tolerability of autologous RBCs coupled with seven MS immunodominant peptides from three myelin proteins and indicated mechanistic evidence for immune tolerance induction. In this study, we describe that pcRBC are taken up in the liver and spleen of mice and humans and are processed by MARCO^+^ macrophages of the liver, i.e., bona fide Kupffer cells (KC), and the marginal zone macrophages of the spleen (*26*).

While growing clinical evidence supports the strategy of using pcRBC instead of nucleated cells for inducing tolerance, the cellular and molecular mechanisms governing antigen processing by tissue-resident phagocytes and the downstream immunological pathways leading to tolerance remain incompletely understood. In the present study, we aimed to elucidate the mechanisms of pcRBC-induced phagocytosis, with a primary focus on the liver, to identify the phagocytic populations involved, and to describe the downstream immunological effects that lead to antigen-specific tolerance induction.

## RESULTS

### Tolerization with pcRBC ameliorates EAE

To assess the efficacy of pcRBC in preventing CNS autoimmunity, we used the well-established EAE model in C57BL/6 mice. Induction of antigen-specific immune tolerance was tested by intravenous injection of syngeneic RBCs coupled with either the encephalitogenic MOG_35–55_ peptide (pcRBC) or the ovalbumin (OVA)_323–339_ control peptide [pcRBC(OVA)]. Seven days later, EAE was induced with the encephalitogenic peptide MOG_35–55_ in complete Freund’s adjuvant (CFA), followed by two doses of pertussis toxin (PTX) (Fig. 1A). MOG_35–55_– (pcRBC) but not OVA_323–339_–coupled RBCs [pcRBC(OVA)] strongly reduced the development of disease symptoms (Fig. 1B and fig. S1, A, C, and D) and increased the probability of survival (fig. S1B). pcRBC(OVA)-treated mice showed EAE scores comparable to the control group, indicating the antigen specificity of our approach. The therapeutic effect of pcRBC was dependent on cell dose with lower disease scores and increased probability of survival for the group receiving the highest dose of 2 × 10^9^ pcRBC compared to the group receiving ≤1.5 × 10^9^ pcRBC (Fig. 1C and fig. S1, F to H). Statistical analysis of the area under the curve (AUC) data confirmed that the 2 × 10^9^ pcRBC group but not the ≤1.5 × 10^9^ pcRBC group showed significant disease amelioration compared to the control group (fig. S1E).

**Fig. 1.**
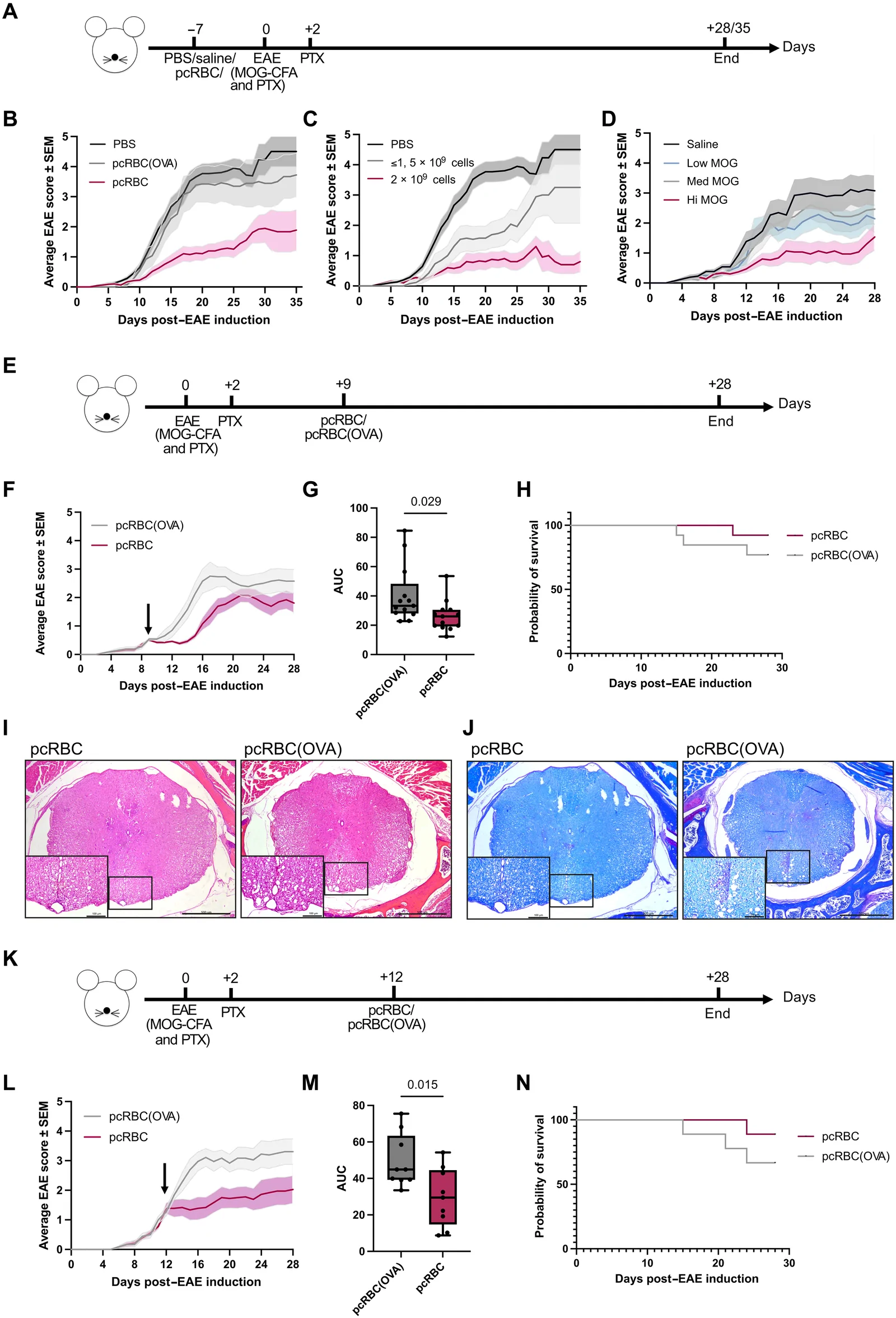
Tolerance induction through pcRBC treatment reduces CNS autoimmune disease. (**A** to **D**) Prophylactic pcRBC treatment. (A) Experimental scheme. (B) Antigen specificity experiment. Clinical scores of pcRBC, pcRBC(OVA), or phosphate-buffered saline (PBS)/saline-treated animals over time (mean ± SEM). Data from six independent experiments [*n* = 13 for pcRBC(OVA), *n* = 33 for pcRBC, and *n* = 28 for PBS/saline] (C) Cell dose titration. Clinical scores of animals treated with ≤1.5 × 10^9^ or 2 × 10^9^ pcRBC or PBS (control) over time (mean ± SEM). Data from six independent experiments (*n* = 13 for ≤1.5 × 10^9^ group, *n* = 20 for 2 × 10^9^ group, and *n* = 28 for PBS/saline). (D) Peptide titration. Clinical scores of animals treated with pcRBC coupled to high (0.9 mg/ml), medium (0.5 mg/ml), or low (0.1 mg/ml) MOG_35–55_ or saline (control) over time (mean ± SEM). Data from three independent experiments (*n* = 13 for saline, *n* = 15 for hi MOG, and *n* = 14 for med and low MOG, respectively). (**E** to **J**) pcRBC treatment at disease onset. (E) Experimental scheme. (F) EAE clinical scores over time (mean ± SEM). (G) AUC comparison of clinical scores; an unpaired Welch’s *t* test was applied. (H) Kaplan-Meier survival curves. Data from three independent experiments (*n* = 13 per group). [(I) and (J)] Histological analysis of spinal cord sections at day 28 post–EAE induction. Representative images of hematoxylin and eosin (H&E) (I) and Luxol Fast Blue (J) staining of pcRBC- or pcRBC(OVA)-treated mice. pcRBC mouse: score 1; pcRBC(OVA) mouse: score 3. Inserts show detailed views of immune infiltrations or lack thereof. Scale bars, 500 μm (40× overview images) and 100 μm (200× inserts). Data representative of one experiment (two mice per group). (**K** to **N**) pcRBC treatment at established disease. (K) Experimental scheme. (L) EAE clinical scores over time (mean ± SEM). (M) AUC comparison of clinical scores; an unpaired Welch’s *t* test was applied. (N) Kaplan-Meier survival curves. Data from two independent experiments (*n* = 9 per group). Statistical significance for relevant panels: *P* < 0.05.

Next, we titrated the peptide concentration used to pulse RBCs, maintaining the identified optimal cell dose. Seven days before EAE induction, C57BL/6 mice were injected intravenously with 2 × 10^9^ pcRBC coupled with three different peptide concentrations (hi: 0.9 mg/ml, med: 0.5 mg/ml, and low: 0.1 mg/ml) or saline as control. The group that received pcRBC^hi^ showed the strongest amelioration of disease, with intermediate improvement in the pcRBC^med^ and pcRBC^low^ groups (Fig. 1D and fig. S1, K and L). The difference in effect size was supported by a statistically significant difference in the AUC values between the pcRBC^hi^ and control groups (fig. S1I). In addition, the pcRBC^hi^ group showed the highest survival rate (fig. S1J). Together, these results indicate that tolerogenic pcRBC treatment is both cell and peptide dose dependent. Accordingly, the highest cell and peptide doses were used in all subsequent experiments.

We next modified our approach to resemble a clinically relevant setting of treating established disease and assessed the efficacy of pcRBC when administered therapeutically. We induced EAE and, once all mice exhibited initial signs of paralysis [day 9 postinduction (p.i.)], administered pcRBC or pcRBC(OVA) as control (Fig. 1E). In this therapeutic setup, treatment with pcRBC again led to a strongly improved disease presentation compared with pcRBC(OVA)-treated mice until day 14 p.i. Thereafter, scores remained lower than in the control group, albeit to a lesser extent (Fig. 1F and fig. S2). The summarized disease activity analysis through AUC confirmed a statistically significant reduction (Fig. 1G), highlighting the therapeutic potential of our tolerogenic approach. Again, the pcRBC-treated group exhibited a higher survival rate (Fig. 1H). Histological analysis of spinal cord sections at day 28 revealed reduced lymphocyte infiltration and diminished tissue swelling in the pcRBC group, as demonstrated by hematoxylin and eosin (H&E) staining (Fig. 1I). Moreover, Luxol Fast Blue staining indicated reduced levels of demyelination in pcRBC-treated mice compared with controls. Hence, both histological analyses showed reduced CNS pathology in pcRBC-tolerized mice (Fig. 1J). To further challenge the therapeutic efficacy of pcRBC treatment, we treated at an established stage of disease. We injected mice with pcRBC or pcRBC(OVA) as control when a clinical score of 1.5 was reached by at least one mouse per group (Fig. 1K). In addition in this setting, pcRBC treatment ameliorated disease symptoms compared to the pcRBC(OVA) group, and this effect was maintained throughout the course of disease (Fig. 1L and fig. S3), again confirmed statistically in the AUC and survival analyses (Fig. 1, M and N). Together, we demonstrate that pcRBC-induced tolerance is antigen specific and dependent on cell and peptide dose. Furthermore, this treatment ameliorates disease in a therapeutic setting.

### Dynamic response of the liver myeloid compartment to pcRBC exposure

We have previously shown that pcRBC are phagocytosed by F4/80^+^MARCO^+^ cells in the liver and spleen, indicative of KC and marginal zone macrophages, respectively (*26*). To assess changes in hepatic phagocytic and other immune populations in an unbiased way, we performed single-cell RNA sequencing (scRNA-seq) of liver CD45^+^ cells in two independent experiments. The experimental groups received either saline or pcRBC, and hepatic cells were prepared for analysis 2 or 17 hours later (fig. S4A). The quality of the cell preparation and CD45 positivity were verified by flow cytometry (figs. S4, B and C, and S5). The sequencing data underwent quality control (QC) and preprocessing. The scRNA-seq profiles from saline-treated animals were comparable between the two time points (fig. S6A). Hence, only the 2-hour saline sample was chosen as the reference control for our analysis. Unsupervised clustering of liver CD45^+^ cells following uniform manifold approximation and projection (UMAP) dimensionality reduction revealed 12 distinct cell clusters (fig. S6B and table S3), including lymphocytic and phagocytic populations annotated by known markers (Fig. 2A). We visualized expression levels and frequencies of cluster-defining genes in a dot plot (fig. S6C). To better understand the consequences of pcRBC uptake, we reclustered the liver’s myeloid cells. The resulting 10 clusters of annotated cells included KC, liver capsular macrophages (LCMs) (*27*), dendritic cells (DCs), plasmacytoid DCs (pDCs), Ly6C^−/+^ monocytes (monoLy6C^−/+^), three clusters of neutrophils, and two clusters of putatively monocyte-derived macrophages (moMF) (Fig. 2B) (*28*). The frequency of neutrophils transiently spiked at 2 hours after pcRBC application and returned to almost baseline levels by 17 hours postinjection. In contrast, the expansion of the two moMF populations was most pronounced at 17 hours. The relative frequency of KC and DCs was markedly reduced at 2 hours and recovered partially at 17 hours (Fig. 2C). The expression levels and detection frequencies of cluster-defining genes are shown in Fig. 2D. The two moMF clusters expressed monocyte-associated markers such as *Ccr2* and *Ly6c1/2* (primarily moMF.1). They also shared similar expression patterns with KC, including *ApoE*, *Adgre1* (F4/80 protein), *Itgam* (CD11b protein), *Fcgr1 and Fcgr3* (Fc gamma receptor), and *Mertk* (MER proto-oncogene tyrosine kinase). However, several other core KC-specific transcripts, such as *Clec4f*, *Marco*, *Vsig4*, *Timd4*, *Cd5l*, and *Fcna* (ficolin 1), were exclusively expressed in the KC cluster (Fig. 2D, fig. S6D, and table S4). Among the two moMF clusters, moMF.2 resembled KC more closely (Fig. 2D and fig. S6D). Consistent with these transcriptional findings, TIM4 expression at the protein level was likewise restricted to the KC and absent from moMF, further supporting the distinction between resident KC and infiltrating moMF, consistent with previous reports (fig. S7) (*29*). Hence, these findings suggest that both clusters represent infiltrating moMF progressing in an ordered differentiation trajectory toward KC-like cells (*29*, *30*).

**Fig. 2.**
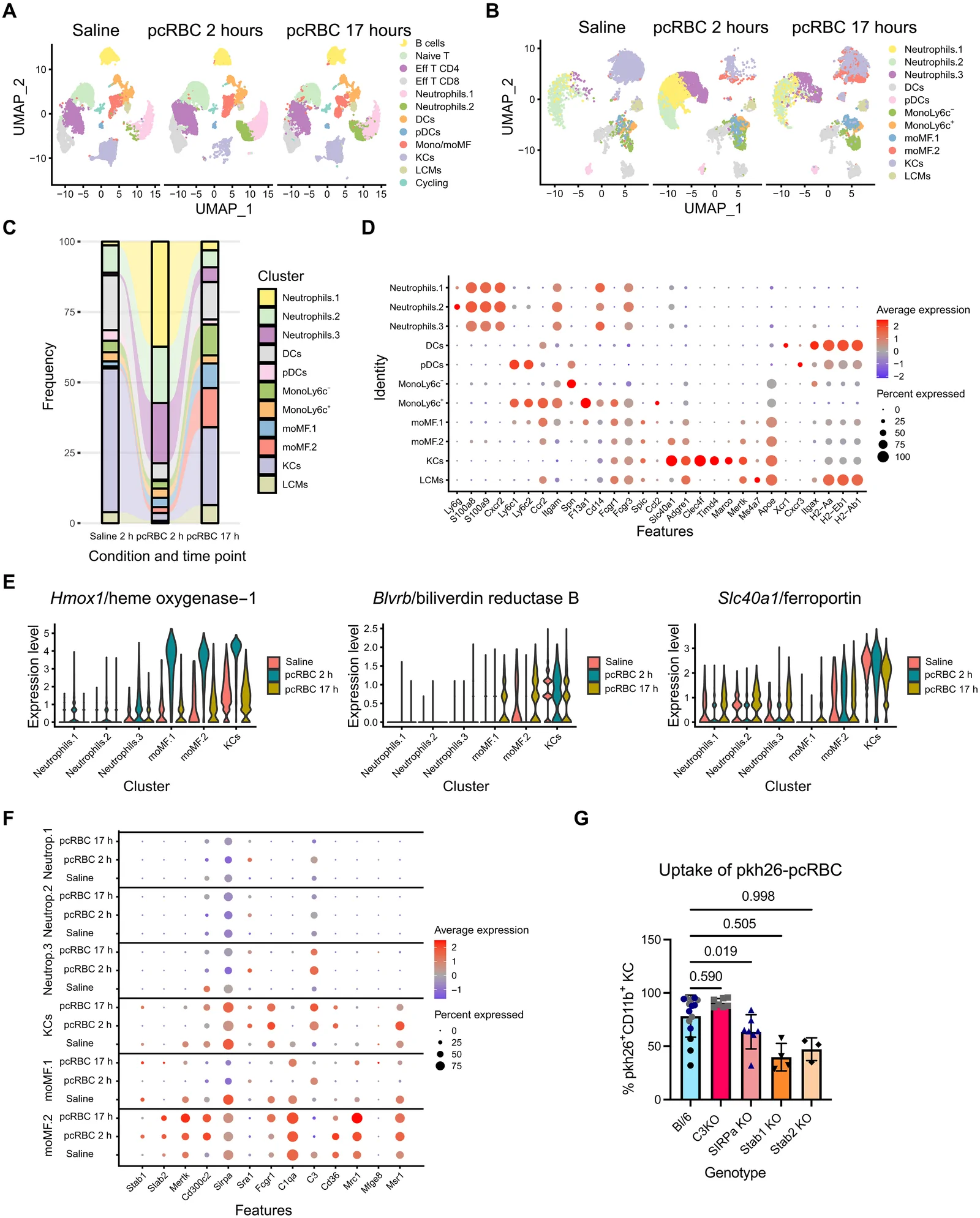
Transcriptional profiling of liver CD45^+^ cells following pcRBC treatment. Wild-type (WT) mice were intravenously injected with 2 × 10^9^ pcRBC coupled to MOG_35–55_ (0.9 mg/ml) or saline and analyzed 2 or 17 hours later by scRNA-seq. (**A**) UMAP showing 12 clusters of CD45^+^ liver cells in saline (2 hours), pcRBC (2 hours), and pcRBC (17 hours) (details in the “Integration and clustering” section). (**B**) UMAP of 10 myeloid clusters after reclustering the myeloid cells only. (**C**) Stacked bar graph of myeloid cluster distribution across conditions. h, hours. (**D**) Dot plot of cluster-defining gene expression and detection frequency in myeloid subsets. (**E**) Violin plots of *Hmox1/*heme oxygenase–1, *Blvrb/*biliverdin reductase B, and *Slc40a1/*ferroportin expression in neutrophils, moMF, and KC across conditions. (**F**) Dot plot of selected phagocytosis-related genes in neutrophils, KC, and moMF across conditions. (**G**) pcRBC uptake in mice deficient for complement component 3 (C3), SIRPα, Stabilin 1, Stabilin 2, and WT controls. Mice received pkh26-labeled pcRBC or saline; liver cells were analyzed by flow cytometry 2 hours later. Gating was performed according to fig. S7A. Bar graph shows %pkh26^+^CD11b^+^ KC (mean ± SD). The C57BL/6 group represents the pooled control mice of all experiments. Data pooled from two to three experiments (*n* = 3 to 15 per group). A linear mixed model was used with batch included as a random effect (batch defined as knockout (KO) genotypes processed alongside the respective WT controls). Group comparisons were performed on estimated marginal means (emmeans package). *P* values were adjusted for multiple comparisons with Benjamini-Hochberg (*P* < 0.05).

As erythrophagocytosis occurs physiologically throughout life and continuously engages the heme catabolism and iron recycling pathways, we assessed whether administration of pcRBC altered the expression of genes involved in these processes. We observed that *Hmox1* (heme oxygenase–1), a major enzyme in heme catabolism, mRNA was highly induced in all KC and in both moMF clusters 2 hours after pcRBC exposure. *Slc40a1* (ferroportin) was constitutively expressed at high levels in KC and at low levels in moMF.2 but was undetectable in moMF.1. Its low or absent expression in the neutrophil clusters remained unchanged upon pcRBC exposure. *Blvrb* (biliverdin reductase B) showed an expression peak in KC after 2 hours and was up-regulated to a lesser extent in both moMF populations at 2 and 17 hours (Fig. 2E). However, given the constitutively high expression of *Hmox1* in KC (*31*), we were unable to discriminate KC that phagocytosed pcRBC from those that had not. These transcriptional responses demonstrate that uptake of a large number of pcRBC transiently induces physiological heme catabolic programs in liver phagocytes, specifically in KC and moMF, which appear to be the main hepatic populations responsible for heme degradation following pcRBC administration.

To identify the receptor systems responsible for pcRBC uptake, we assembled a gene list from the literature (*32*, *33*) and assessed their expression in our dataset (Fig. 2F). Two hours after pcRBC injection, we observed a strong induction of *C3* (complement), *Msr1* (macrophage-scavenger receptor, CD204), and *CD36* (a scavenger receptor) in KC, in addition to a more modest increase in *Fcgr1* (Fcγ receptor). In moMF.2, the strongest candidates appeared to be *Mertk* (a tyrosine kinase), *CD300* (a surface receptor family), *C1qa* (complement), *CD36*, and *Mrc1* (mannose receptor C-type 1/CD206) (Fig. 2F). To test their functional relevance, we examined pcRBC uptake in mice deficient in *C3*, *SIRPα*, *Stb1*, *or Stb2* (stabilins). Pkh26-labeled pcRBC were injected into gene-deficient and wide-type (WT; control) animals, and hepatic phagocytes were analyzed 2 hours after pcRBC injection by flow cytometry. pcRBC uptake by KC was significantly lower in SIRPα-deficient mice compared with WT controls, whereas no differences were observed in complement component 3 (C3), Stabilin 1, or Stabilin 2 knockout (KO) mice. Given the established inhibitory role of SIRPα in phagocytosis (*34*), this reduction is opposite to the expected outcome, suggesting potential compensatory mechanisms resulting from the constitutive KO model (Fig. 2G and fig. S8).

Our scRNA-seq analysis revealed a dynamic response of the hepatic myeloid compartment. The KC population was largely replaced by other infiltrating myeloid cells, such as neutrophils and moMF, but only KC and moMF showed heme-degrading activity. moMF.1 and moMF.2 likely reflect sequential developmental stages along a monocyte-to-KC transition trajectory. While we excluded a major role of C3, SIRPα, and Stabilin 1 and 2 in the uptake of pcRBC experimentally, our expression profile indicated multiple alternative candidate receptors, including *Msr1*, *Cd36*, *Mertk*, and members of the *Cd300* family.

### KC are the major pcRBC-phagocytosing population in the liver

To confirm the above response of the hepatic myeloid cell compartment to pcRBC exposure, we used flow cytometric analysis of liver and spleen 2 and 17 hours after injection of 2 × 10^9^ pcRBC or 0.9% saline (control) into WT C57BL/6J mice (fig. S4A). At 2 hours, we confirmed the decreased frequency (approximately 2.5 times) of tissue-resident KC (CD45^+^F4/80^hi^CD11b^+^ cells) (Fig. 3A and gating strategy shown in fig. S9A), which corresponded to a 50% decrease in absolute cell numbers (Fig. 3B and fig. S9B). We demonstrate a substantial increase (∼4 times) in the frequency and cell number of F4/80^−^CD11b^+^ cells, a population that includes monocyte-derived cells and neutrophils (here referred to as Mono/Neut) (Fig. 3B and fig. S9B). A F4/80^low^CD11b^+^ population, corresponding to moMF identified in our scRNA-seq data, appeared at 2 hours, with its frequency increasing over time (17 hours). KC frequencies remained low at 17 hours, while Mono/Neut decreased after peaking at 2 hours (Fig. 3B). Consistent with our scRNA-seq observation, the infiltrating Mono/Neut population at 2 hours consisted predominantly of recently infiltrating neutrophils (CD45^+^F4/80^−^CD11b^+^Ly6C^+^Ly6G^+^ cells), which decreased in frequency at 17 hours postinjection. In contrast, infiltrating monocytes (CD45^+^F4/80^−^CD11b^+^Ly6C^+^Ly6G^−^ cells) composed most Mono/Neut subset at this later time point, indicating distinct temporal patterns of circulating cell infiltration into the liver after pcRBC injection (Fig. 3, C and D).

**Fig. 3.**
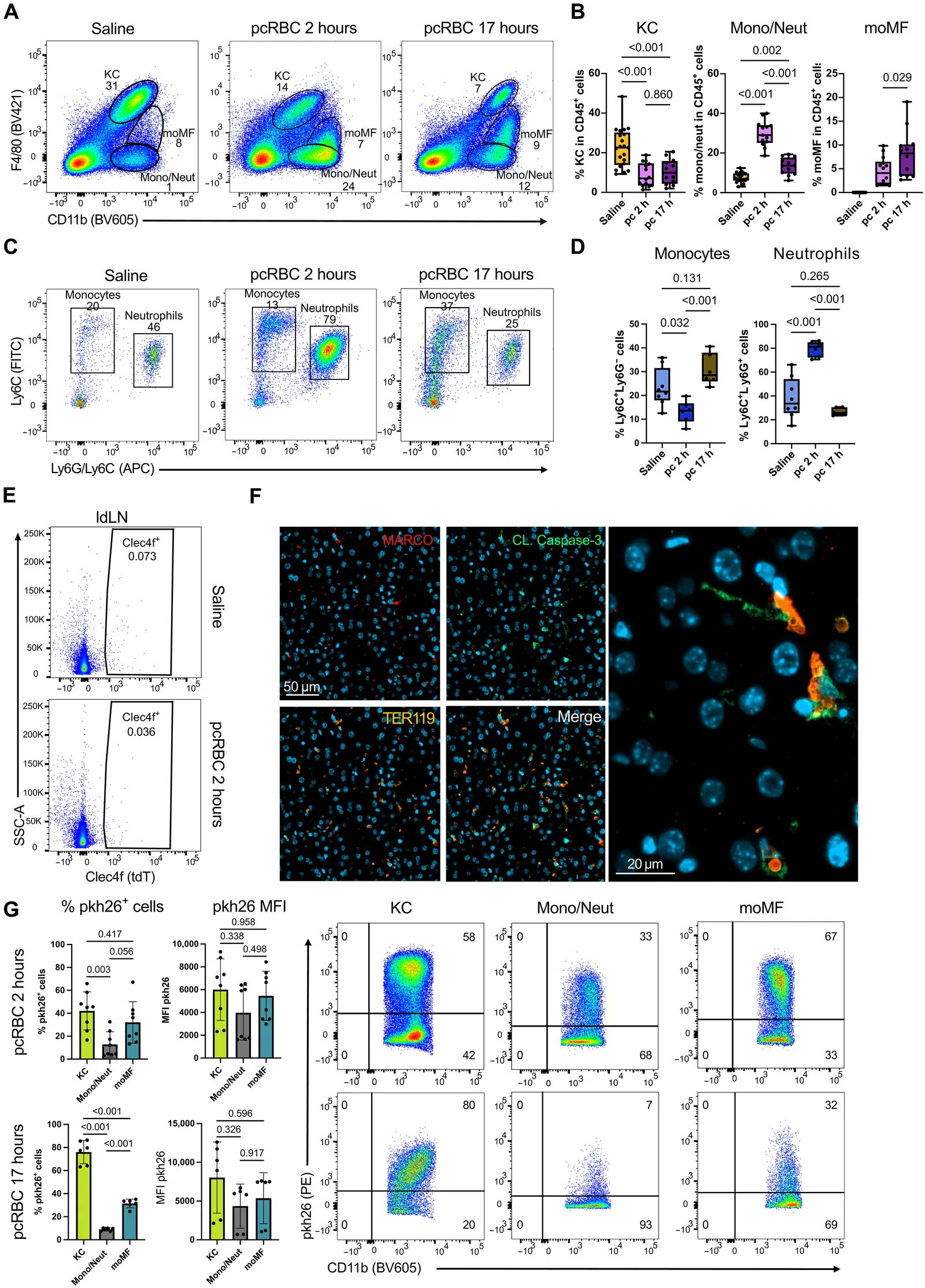
pcRBC treatment alters hepatic myeloid cell composition. WT mice were intravenously injected with 2 × 10^9^ pcRBC coupled to MOG_35–55_ (0.9 mg/ml) or saline and analyzed 2 or 17 hours postinjection as described in Materials and Methods. (**A**) Representative fluorescence-activated cell sorting (FACS) plots of liver nonparenchymal cells (NPC), depicting KC, Mono/Neut, and moMF in saline versus 2 and 17 hours post–pcRBC injection. (**B**) Frequencies of KC, Mono/Neut, and moMF across time points. Pooled data from eight independent experiments (*n* = 12 to 22 per group). (**C**) Representative FACS plots of liver Mono/Neut populations in saline versus 2 and 17 hours post–pcRBC injection. Saline group (control) in (A) to (C) represents data from 2 and 17 hours post–saline injection. (**D**) Frequencies of monocytes and neutrophils; two experiments [*n* = 8 (saline) and *n* = 6 (pcRBC; 2 and 17 hours)]. (**E**) FACS plots of liver-draining lymph node (ldLN) CD45^+^ cells (normalized) 2 hours posttreatment; *n* = 2 (saline) and *n* = 3 (pcRBC). (**F**) High-dimensional microscopy of liver sections obtained 2 hours post–pcRBC treatment, stained for MARCO (KC; red), TER119 (RBCs; orange), and cleaved Caspase-3 (green); one experiment (*n* = 2 per group). CL., cleaved. (**G**) Bar plots showing frequencies and mean fluorescence intensity (MFI; left), and representative FACS plots (right) of pkh26^+^CD11b^+^ KC, Mono/Neut, and moMF; representative of two to four experiments (*n* = 8 for 2 hours and *n* = 6 for 17 hours). Statistical analysis: one-way analysis of variance (ANOVA) with multiple comparisons [KC frequency (B); monocyte frequency (D); pkh26^+^CD11b^+^ frequency (2 hours) (G)], unpaired *t* test [moMF frequency (B)], and Brown-Forsythe and Welch ANOVA (others). Statistical significance: *P* < 0.05.

Next, we sought to identify the underlying mechanism behind the decreased KC numbers after pcRBC administration. Previous studies using electron microscopy (*35*) and in vivo imaging (*36*) have shown that KC in rats and mice, respectively, can migrate to the liver-draining lymph nodes (ldLN). To assess possible migration of KC to ldLN in our model, we used a mouse line expressing the red fluorescent protein tdTomato (tdT) under the control of the KC-specific *Clec4f* promoter (*Clec4f*-Cre-tdTomato mice) (*37*). We injected these *Clec4f*-Cre-tdTomato mice intravenously with 2 × 10^9^ pcRBC or 0.9% saline and assessed the presence of *Clec4f*-expressing tdT^+^ cells (KC) in ldLN, inguinal LN, and spleen 2 hours later by flow cytometry. Analysis of the hepatic CD45^+^ fraction confirmed the reduction in KC abundance (fig. S9C). However, no tdT^+^ cells were detected in any of the three examined organs (Fig. 3E and fig. S9C), excluding extensive KC emigration in response to pcRBC treatment.

To assess whether cell death rather than migration contributed to the reduction in KC after massive erythrophagocytosis, as previously reported for red pulp macrophages (*38*), we performed a multiplex immunofluorescence analysis on liver sections 2 hours after pcRBC injection. Thorough perfusion of the livers before fixation was performed to eliminate circulating RBCs. Staining for TER119 confirmed the uptake of pcRBC by resident KC (MARCO^+^ cells). These cells also showed staining for cleaved Caspase-3, indicating that decreased KC numbers observed post–pcRBC injection can be attributed to apoptosis (Fig. 3F).

To determine which hepatic cell types contribute to pcRBC clearance more precisely, we injected mice with pcRBC labeled with the membrane dye pkh26. Flow cytometry analysis of hepatic myeloid cells at 2 and 17 hours postinjection showed uptake of pcRBC by all three key myeloid populations. In accordance with our scRNA-seq analysis, KC and newly differentiated macrophages carried the highest load of pcRBC at 2 hours postinjection (42 and 32%, respectively) (Fig. 3G).

At 17 hours, most pkh26 staining was contained in KC (76%), with significantly lower levels in Mono/Neut (9%) and moMF (32%) populations (Fig. 3G). In line with this, monocytes and neutrophils showed relatively low pkh26-pcRBC uptake, with 10 and 16% at 2 hours and 15 and 19% at 17 hours, respectively (fig. S10A). Preliminary analysis of liver sinusoidal endothelial cells (LSECs) demonstrated that a small fraction of CD45^–^CD31^+^ and CD45^–^CD31^+^LYVE1^+^ LSECs was pkh26^+^, indicating the presence of pcRBC-derived membrane material within these cells (fig. S10B). We further confirmed specific pcRBC uptake by intracellular TER119 staining and by cytoplasmic dye CellTrace Far Red (CTFR)–labeled pcRBC (fig. S11, A and B). Among the different labeling approaches, pkh26 proved the most sensitive readout in depicting the uptake of pcRBC and was used in subsequent experiments.

To test whether the efficient uptake of RBCs by liver phagocytes, most prominently KC, was dependent on alterations in the membrane or metabolism of RBCs induced by the coupling process, we injected mice with either fresh RBCs or pcRBC and analyzed liver nonparenchymal cells (NPC) postinjection as before. The uptake of pcRBC and the changes in the myeloid cell composition were specific to pcRBC, as injection of fresh uncoupled RBCs did not lead to any notable changes in the myeloid subsets of the liver (fig. S12A).

While our primary analysis focused on liver phagocytes, we also assessed the myeloid cell composition and pcRBC uptake in the spleen. Similar to the liver, circulating Mono/Neut infiltrated the spleen; however, no moMF population emerged (fig. S12, B and C). pcRBC were taken up by splenic F4/80^+^CD11b^−^ macrophages and F4/80^−^CD11b^+^ cells (fig. S12D). In summary, pcRBC phagocytosis by KC triggers their apoptotic loss and concurrently drives monocyte-to-moMF differentiation, resulting in sustained changes in hepatic myeloid cell subset composition.

### pcRBC administration induces a tolerogenic KC phenotype

Given the prominent role of KC in pcRBC uptake and their generally recognized tolerogenic and anti-inflammatory properties (*39*), we explored by flow cytometry how pcRBC phagocytosis affects the KC phenotype. To this end, we compared KC that had phagocytosed pkh26-labeled pcRBC (pkh26^+^ KC) with those that had not (pkh26^–^ KC). pkh26^+^ KC showed increased PD-L1 expression as early as 2 hours post–pcRBC injection compared to pkh26^–^ KC (Fig. 4A, left). This effect persisted at 17 hours posttreatment (Fig. 4A, middle) and was statistically significant for both time points (Fig. 4A, right). A similar increase was also observed in pcRBC-phagocytosing moMF (fig. S13).

**Fig. 4.**
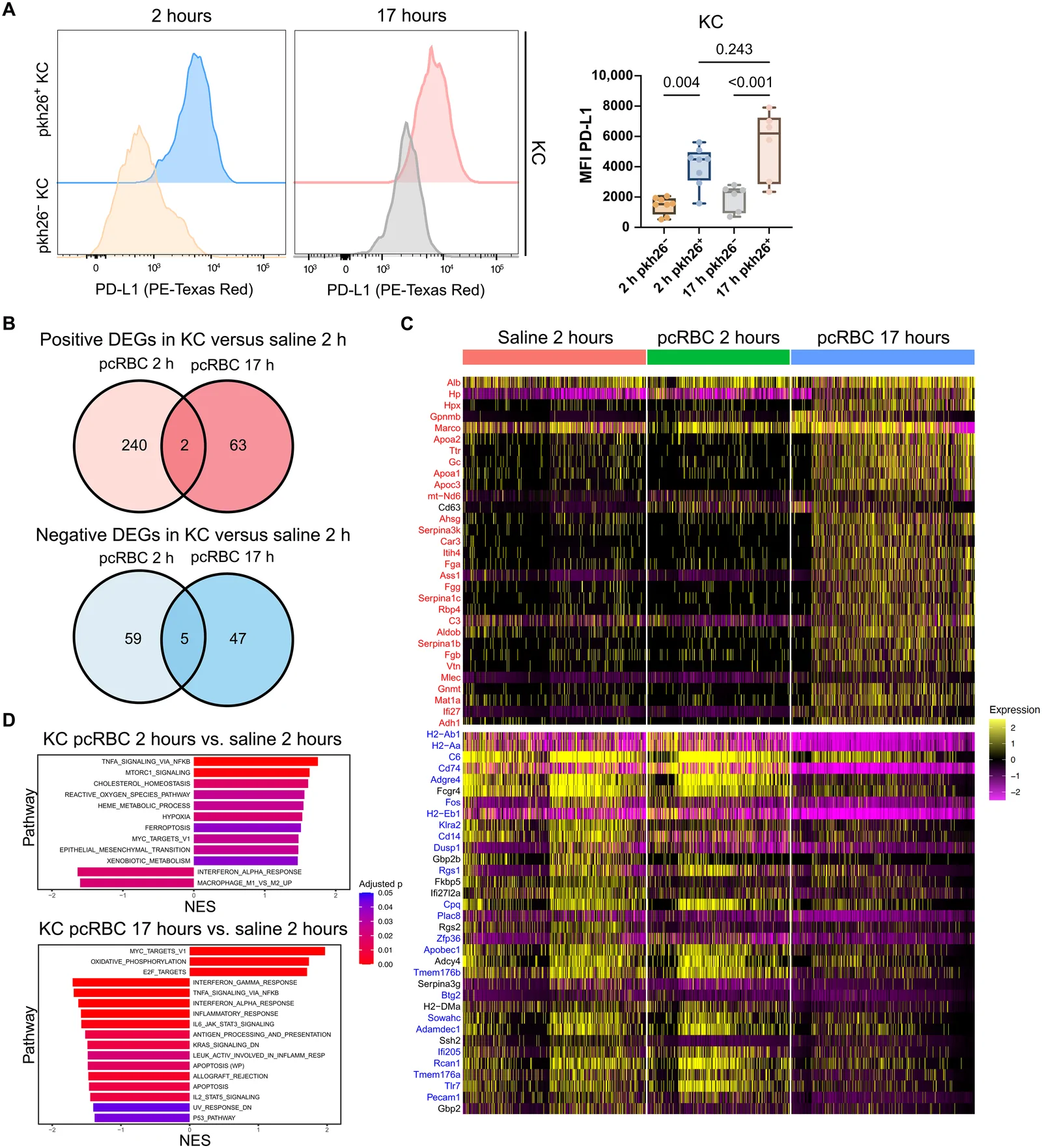
pcRBC treatment induces a tolerogenic transcriptional program in KC. WT mice were intravenously injected with 2 × 10^9^ pcRBC coupled to MOG_35–55_ (0.9 mg/ml) or saline and analyzed by scRNA-seq at 2 or 17 hours postinjection. (**A**) Representative histograms of PD-L1 expression in pkh26^–^ and pkh26^+^ KC 2 hours (left) and 17 hours post–pcRBC treatment (middle) and PD-L1 MFI (right). Data from three independent experiments for 2 hours (*n* = 8) and two independent experiments for 17 hours (*n* = 6). Ordinary ANOVA with multiple comparisons was performed. Statistical significance: *P* < 0.05. (**B**) Venn diagrams of differentially expressed genes (DEGs) in KC comparing pcRBC (2 hours) versus saline (2 hours) and pcRBC (17 hours) versus saline (2 hours). Up-regulated (top) and down-regulated (bottom) gene overlaps are shown. (**C**) Heatmap of the top 30 DEGs in KC at 17 hours post–pcRBC injection versus saline (2 hours). Genes also differentially expressed between pcRBC 17 hours and 2 hours are highlighted (red = up, blue = down). (**D**) Gene set enrichment analysis (GSEA) of KC comparing pcRBC (2 hours) versus saline (2 hours) (top) and pcRBC (17 hours) versus saline (2 hours) (bottom); pathways ranked by normalized enrichment score (NES). scRNA-seq data from two independent experiments (*n* = 2 per group per time point). DEGs defined by false discovery rate (FDR) < 0.01 and |logFC| ≥ 1.5; pathway significance at FDR < 0.05.

We next used our scRNA-seq dataset to further characterize the transcriptional changes in KC post–pcRBC injection. *Hmox1* is constitutively expressed at high levels in KC (*31*) and, as shown above, was increased by all KC post–pcRBC injection. Thus, our scRNA-seq dataset did not allow discrimination between KC that had phagocytosed pcRBC and those that had not. We identified 306 and 117 genes that were differentially expressed at 2 and 17 hours post–pcRBC injection, respectively, compared to saline controls. Minimal overlap was observed between the two time points (two up-regulated and five down-regulated), suggesting time-specific transcriptional profiles that likely reflect a functional shift in KC after pcRBC uptake (Fig. 4B and table S5). The most pronounced transcriptional changes occurred at 2 hours (fig. S14, A and B, and table S6), with *S100a8* and *S100a9*, alarmins driving leukocyte recruitment (*40*), being the strongest up-regulated genes at 2 hours in a small subset of KC (fig. S14A). Notably, *Ccl2* and *Ccl3*, described to mediate monocyte recruitment to the liver after stressed RBCs uptake (*29*), and *Spic*, a heme-induced transcription factor typically expressed in iron-recycling spleen and bone marrow macrophages (*41*), were also up-regulated at 2 hours post–pcRBC injection (fig. S14A and table S6).

*Alb*, a hepatocyte-specific marker (*31*), was the most strongly up-regulated gene in KC relative to saline-treated controls, 17 hours post–pcRBC injection (Fig. 4B). While KC are not known to express *Alb* (*42*), *Alb* expression was restricted to the KC cluster (fig. S14C), arguing against ambient RNA contamination. At the same time, *Marco* ranked fifth among up-regulated genes. This scavenger receptor is expressed in tolerogenic mouse bone marrow–derived macrophages and is required for tolerance induction via antigen-coupled microparticles (*43*). Other up-regulated genes included those associated with metabolic functions and immunomodulation (e.g., *Ass1*, *Apoa1/2*, *Apoc3*, *Hp*, *Hpx*, *C3*, *Serpina* family, and *Gpnmb*). All these genes were also up-regulated in the 17 hours versus 2 hours post–pcRBC injection comparison, supporting a time-specific transcriptional modulation in KC as mentioned above. In contrast, key major histocompatibility complex class II (MHC-II) components (*Cd74*, *H2-Aa*, *H2-Ab1*, *H2-Eb1*, and *H2-DMa*) and a group of interferon-induced genes (*Ifi27l2a*, *Ifi205*, and *Gbp2b*) were down-regulated, indicating a reduction in antigen presentation and immune activation in KC post–pcRBC injection (Fig. 4C and table S6). Notably, comparison of the two post-saline time points revealed minimal differences, indicating the robustness of our data (fig. S14C and table S6).

Gene set enrichment analysis (GSEA) of KC at 2 hours compared to control revealed up-regulation of genes associated with tumor necrosis factor–α (TNFα) signaling via nuclear factor κB, as well as genes involved in cholesterol homeostasis, suggesting early inflammatory and metabolic changes associated with clearance and breakdown of pcRBC. Genes related to heme metabolism, reactive oxygen species (ROS), and ferroptosis were also up-regulated 2 hours post–pcRBC injection, indicating increased oxidative stress after pcRBC injection, leading to cell death via ferroptosis (Fig. 4C, top, and table S7). At 17 hours post–pcRBC treatment, *Myc* and *E2F target* gene sets (*44*, *45*), key regulators of cell cycle progression, were up-regulated, indicating a high proliferative activity in KC consistent with the partial recovery in numbers seen in our scRNA-seq analysis. Concordantly, we found that the p53 pathway, which usually leads to cell cycle arrest (*46*), was strongly down-regulated. The induction of the *Oxidative Phosphorylation* gene set suggests increased metabolic activity of KC post–pcRBC exposure. Down-regulated pathways at 17 hours after pcRBC application were dominated by immune-related gene sets, such as interferon-α (IFN-α) and IFN-γ responses, TNFα signaling, inflammatory responses, allograft rejection, leukocyte activation in inflammatory responses, and antigen processing and presentation. This indicates a general shift in the cell program away from inflammation, thus possibly supporting tolerance. The assessed *Tolerance Induction* gene set was, unexpectedly, among the negatively enriched pathways (Fig. 4C, bottom, and table S8), albeit with a rather high *P* value. This tolerance induction pathway (GO:0002507) is composed of genes expressed not exclusively by KC but also by other immune cells, including T and B cells, possibly accounting for the observed negative enrichment of this pathway in KC. Thus, additional data may be warranted here (table S9). Overall, our findings suggest that following pcRBC uptake, KC progressively acquire a tolerogenic phenotype characterized by reduced immune and inflammatory responses, increased PD-L1 expression, and enhanced metabolic and proliferative activity.

### KC are indispensable for pcRBC-mediated tolerance induction in EAE

The above experiments demonstrated a major contribution of KC in pcRBC clearance, followed by substantial changes in the size and functional parameters of the KC population; however, they did not address the role of KC in the pcRBC-mediated tolerization directly. To assess their role in tolerance induction in our model more directly, we selectively depleted KC in vivo. We intercrossed *Clec4f*-Cre-tdTomato with the iDTR mouse line (*47*) to generate *Clec4f*-Cre-tdTomato/iDTR (here referred to as KC-DTR) double-mutant line, in which KC specifically express diphtheria toxin (DTx) receptor (DTR) upon Cre-mediated recombination (*37*). First, we confirmed the ablation of KC 12 hours post–DTx [100 ng intraperitoneally (ip)] injection by flow cytometry (Fig. 5A) and fluorescence microscopy (Fig. 5B). We observed the previously described reactive infiltration of blood monocytes into the liver (*30*, *37*) and confirmed the reported repopulation with KC 7 and 14 days later (fig. S15A) (*30*).

**Fig. 5.**
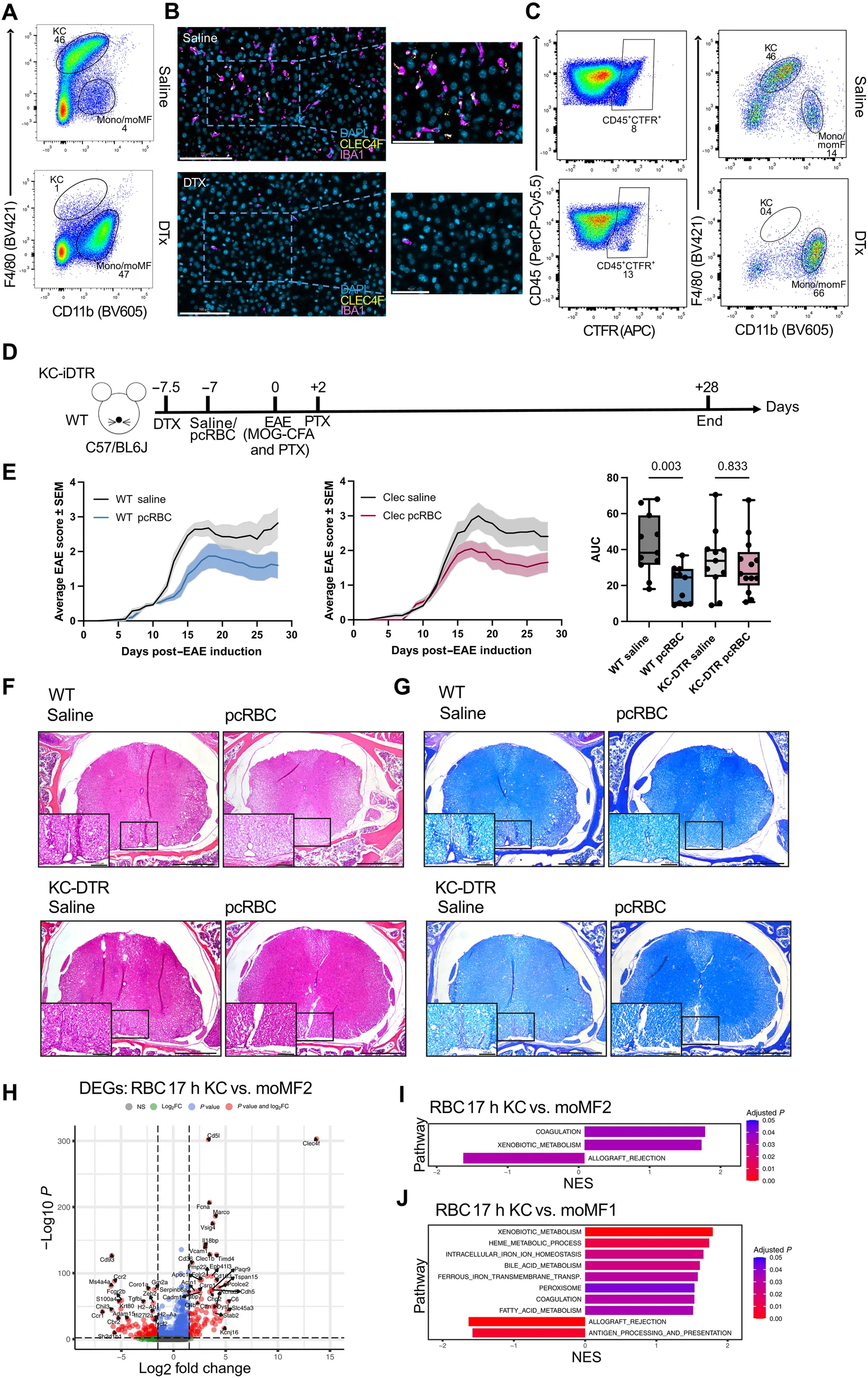
KC are required for pcRBC-mediated tolerance induction in EAE. (**A**) Representative FACS plots of liver myeloid cells from KC-DTR mice 12 hours post–saline/DTx treatment (*n* = 9). The representative saline plot is also shown in fig. S15A; gating according to fig. S7A. (**B**) High-dimensional microscopy of mouse liver tissue after KC depletion on Comet. 4′,6-diamidino-2-phenylindole (DAPI; nuclei), CLEC4F (KC), and IBA1 (macrophages). Images were acquired at ×20 magnification. Scale bars, 100 μm (overview) and 50 μm (insert). Data representative of one experiment (*n* = 2 per group). (**C**) Flow cytometric analysis of CTFR-labeled pcRBC uptake by liver NPC in KC-intact or KC-depleted mice 2 hours postinjection. CD45^+^CTFR^+^ cells are pregated on single live CD45^+^ cells. Data representative of three independent experiments (*n* = 5 to 8 per group). (**D** to **G**) Prophylactic pcRBC treatment of KC-intact or KC-depleted EAE mice. (D) Experimental scheme. (E) Mean EAE scores and AUC for WT and KC-DTR mice treated with pcRBC or saline; pooled data from three independent experiments (*n* = 11 to 13 per group). One-way ANOVA for AUC comparisons (WT saline versus WT pcRBC and KC-DTR saline versus KC-DTR pcRBC). Statistical significance: *P* < 0.05. [(F) and (G)] Representative images of H&E (F) and Luxol Fast Blue (G) staining of KC-DTR and WT mice treated with pcRBC or saline (control). Clinical scores: WT saline, 3.5; WT pcRBC, 0.5; KC-DTR saline, 2; KC-DTR pcRBC, 1. Inserts show detailed views of immune infiltrates. Scale bars, 500 μm (40× overview) and 100 μm (200× inserts). Data representative of one experiment (two mice per group). (**H**) Volcano plot of DEGs between moMF.2 and KC at 17 hours post–pcRBC injection. Up-regulated genes (red) and down-regulated genes (blue). Dashed lines indicate the significance threshold (*P* < 0.05) and fold change cutoffs (|log_2_FC| > 1.5). NS, not significant. (**I** and **J**) GSEA comparing transcriptional programs in KC versus moMF.2 (I) and moMF.1 (J) at 17 hours post–pcRBC injection. Bar length represents the NES; color indicates *P* value.

Flow cytometry confirmed that within 2 hours of pcRBC injection, only infiltrating monocytes and moMF phagocytosed pRBC in KC-depleted mice (Fig. 5C). Abundance of CTFR-labeled pcRBC in the blood (fig. S15B) was comparable between saline- and DTx-treated KC-DTR mice, indicating that infiltrating monocytes and differentiating moMF in the liver efficiently phagocytose pcRBC in the absence of KC.

To assess the contribution of KC in tolerance induction in our system, we ablated KC in KC-DTR mice using DTx (100 ng, ip) and similarly treated WT mice as controls. Twelve hours later, we injected pcRBC (or saline) for possible tolerization, followed by EAE induction after 7 days (Fig. 5D). DTx-treated WT mice (KC-intact) that received saline developed typical symptoms, while those that had received pcRBC presented with an ameliorated disease course. In contrast, DTx-treated KC-DTR mice (KC-depleted) injected with pcRBC presented with similar disease progression as saline-treated controls (Fig. 5E and fig. S16, A to C). Consistently, AUC analysis of disease scores demonstrated that KC ablation eliminated the disease-modifying effect of pcRBC treatment, with a difference in mean AUC of only 3.4 between KC-DTR saline and KC-DTR pcRBC, compared with 22 for the same comparison in WT mice (Fig. 5E, right). Histological assessment (H&E and LxB staining) of spinal cord sections demonstrated comparable lymphocyte infiltration, tissue swelling, and demyelination levels between pcRBC- and saline-treated mice lacking KC (Fig. 5, F and G). Together, pcRBC application in the absence of KC failed to induce tolerance, thus proving an essential, nonredundant role for KC in the induction of antigen-specific immune tolerance, which cannot be compensated by infiltrating monocytes and moMF.

To identify genes and pathways that may be responsible for the unique tolerogenic properties of KC, we then compared in our scRNA-seq dataset KC to the most KC-like nontolerogenic populations, namely, moMF.1 and 2 (Fig. 2D). KC were defined by *Clec4f* expression and coexpression of *Marco*, *Timd4*, *Cd5l*, *Vsig4*, *Fcna*, and *Cdh5*. Compared to moMF2, KC showed higher expression of *Il18bp*, which ameliorates IL-18 signaling implicated in EAE development (*48*). Similarly, KC also showed higher levels of *Cd5L*, a secreted glycoprotein from the scavenger receptor cysteine-rich superfamily, which is present in nonpathogenic T_H_17 cells and is known to modulate lipid metabolism and macrophage apoptosis (*49*). KC expressed lower levels of antigen-presentation genes (e.g., *H2-Ab1*), immunoglobulin superfamily genes (e.g., *Fcgr2b*), and genes involved in cell migration (e.g., *Ccr2* and *Ccr1*) (Fig. 5H and table S10). Only three gene sets were found to be enriched between moMF.2 and KC. The xenobiotic metabolism pathway was enriched in KC, with leading-edge genes including *Hmox1*, *Cd36*, and *Aldh2*, together with other redox- and detoxification-associated genes, suggesting an increased metabolic, oxidative stress–dampening mechanism of KC. In addition, the allograft rejection pathway was found to be decreased in KC, indicating a lower inflammatory capacity of these cells (Fig. 5I and table S11). In contrast, GSEA analysis between KC and moMF.1 revealed more prominent expression of immunological gene sets that could play a role in tolerance development. These included decreased antigen processing and presentation, allograft rejection, and IFN-α and IFN-γ responses in KC (Fig. 5J and table S12). In this comparison, three RBCs degradation–related gene sets, namely, intracellular iron ion homeostasis, heme metabolic process, and ferrous iron transmembrane transport, were up-regulated in KC. Additional gene sets with altered expression cover catabolic pathways, possibly also linked to the degradation of phagocytosed cellular debris and pcRBC.

Together, the above data establish KC as the population that mediates tolerance induction after administration of pcRBC. KC show notable immune-related transcriptional differences compared to KC-like hepatic myeloid cells, which likely render them critical orchestrators of pcRBC-induced immune tolerance.

## DISCUSSION

Antigen-specific immune tolerance mediated by pcRBC is a promising therapeutic approach for autoimmune diseases, currently investigated in early-phase clinical trials for patients with MS (*26*, *50*). Preclinical studies with pcSplenocytes demonstrated an indirect mechanism of tolerance induction in which peptide-coupled cells undergo apoptosis and are phagocytosed by professional APCs, which present the target antigen in a tolerogenic manner. While studies with pcSplenocytes highlighted the major role of splenic phagocytes (*17*, *18*), preliminary human and mouse data indicated a critical role for the liver in pcRBC clearance (*26*). Accordingly, we aimed to characterize the primary phagocytic cell populations and the mechanism of pcRBC-mediated tolerance within the liver using a mouse model.

In our in-depth study, we confirm that pcRBC-mediated immune tolerance is antigen specific and dependent on both cell and peptide dose, consistent with prior reports using pcSplenocytes (*12*, *13*). Similar protective effects have been reported for neuroantigen-coupled splenocytes (*14*, *16*) and peptide-coupled microparticles (*43*) in SJL/J mice challenged with EAE. Podojil *et al.* (*51*) also showed that pcRBC(MOG) ameliorated EAE in C57BL/6 mice in a prophylactic setting albeit without a systematic characterization of tolerization and no in-depth mechanistic analysis. In addition, Miller and Turley (*52*) cited similar, so-far unpublished evidence in their review.

In our study, we found that a single prophylactic treatment induced long-term immune tolerance, whereas therapeutic intervention after disease onset yielded rapid disease amelioration for 5 days, followed by moderate longer-lasting improvement and at established disease (score: 1.5) a more persistent disease amelioration. The reduced efficacy of pcRBC against established disease compared to prophylactic treatment may reflect two nonmutually exclusive factors: (i) Intervention in an immunologically naïve system with respect to prior exposure to the disease-inducing autoantigen differs fundamentally from postonset therapeutic intervention in terms of the frequency and activation state of antigen-specific T cells, as well as the severity of the neuroinflammatory environment (*53*); or (ii) the establishment of long-term tolerance may depend on Tr1 or regulatory T cell development (*54*), during which time uncontrolled disease progression can exceed beyond effective suppressive capacity. This interpretation is supported by prior studies in SJL mice, where treatment at disease peak prevented further relapse while failing to completely suppress initial manifestation (*16*). We observed that treatment during established disease produced a stronger and more durable clinical benefit than treatment at disease onset. A similar observation has been previously reported by Pishesha *et al.* (*55*), who showed that administration at score of 1 of RBCs carrying antigen linked by sortase resulted in complete remission. The differences in the cell and antigen doses and the coupling mechanism may have contributed to the differences in therapeutic efficacy between the two studies. In addition, Pishesha *et al.* used transgenic RBCs, which precludes transfer of their method into the clinic. Therefore, to this end, the mechanisms underlying the differential efficacy of the treatment on the disease stages in our study remain unclear.

Following intravenous injection, pcRBC accumulated rapidly in the liver and spleen. In the liver, resident KC emerged as the dominant early phagocytes for pcRBC clearance, while monocytes, neutrophils, and moMF were recruited and contributed to pcRBC uptake. This is consistent with studies describing aged and eryptotic RBCs (i.e., erythrocytes undergoing an RBCs-specific programmed cell death–like process) (*56*) uptake by hepatic phagocytes (*57*, *58*). We also observed an involvement of splenic macrophages, as well as monocytes and neutrophils infiltrating the spleen, in pcRBC clearance, which is supported by models of damaged or aged RBCs uptake (*29*, *38*). While scRNA-seq revealed up-regulation of several phagocytosis-related receptors in KC (*C3*, *Msr1*, *Cd36*, and *Fcgr1*) and moMF.2 (*Mertk*, *Cd300*, *C1qa*, *Cd36*, and *Mrc1*), functional depletion studies rendered unlikely a role for C3, SIRPα, and Stabilin 1 and Stabilin 2 in pcRBC phagocytosis, which are typically required for other forms of RBCs clearance (*59*–*61*). Erythrophagocytosis experiments identified CD36 and Msr1 receptors as necessary for the uptake of stressed RBCs (*29*), both of which we found expressed stronger in KC after treatment. Together, these findings suggest that pcRBC uptake follows a distinct phagocytic program that is independent of several classical RBCs clearance mechanisms while potentially engaging receptors associated with the uptake of stressed RBCs.

Exposure to pcRBC resulted in a rapid, transient loss of KC numbers, which we attribute to cell death rather than migration. A similar loss of “stressed” KC has been reported across diverse settings, including liver infections (*62*, *63*), sterile injury, enhanced erythrophagocytosis, and metabolic liver diseases (*64*, *65*). Cleaved Caspase-3 staining in TER119^+^ KC indicates that apoptosis contributes to this early decline in KC numbers. Similarly, Podojil *et al.* demonstrated that phagocytosis of pcRBC induces myeloid cells to undergo apoptotic cell death and release oxidized DNA to promote immune tolerance (*51*). In parallel, enrichment of ROS and oxidative phosphorylation pathways in our scRNA-seq dataset suggests that erythrophagocytosis imposes metabolic and oxidative stress on KC. Although Hmox1-mediated heme degradation generally protects against oxidative damage, hyperactivation of *Hmox1* has been reported to predispose KC to ferroptosis (*66*). Erythrophagocytosis-induced differentiation of tolerogenic MARCO^hi^ MHC-II^lo^ KC has been shown to depend on NRF2-mediated transcriptional reprogramming (*67*), suggesting that heme-driven NRF2 signaling may contribute to tolerogenic KC reprogramming following pcRBC uptake. Following this transient depletion, the recovery of KC is driven by local self-renewal and monocyte recruitment via CCL2, CCL3, and macrophage colony-stimulating factor (*29*), suggesting that the loss of KC is an integral part of immunoregulatory mechanisms in the tolerogenic response to pcRBC. From a therapeutic perspective, although a transient reduction in KC numbers could theoretically affect liver homeostasis and immune surveillance, the rapid restoration of the KC compartment observed in previous studies (*29*) and the favorable tolerability and safety profile observed in patients with treated MS in two independent phase Ib clinical trials (*26*, *50*) suggest that any such effects are likely transient and clinically insignificant.

The requirement of KC for tolerance induction became evident by their conditional depletion. Despite the ability of infiltrating Ly6C^hi^ monocytes to phagocytose pcRBC in the absence of KC, they proved functionally insufficient to mediate pcRBC-induced tolerance. This deficiency likely derives from the specialized tolerogenic properties of resident KC, characterized by IL-10 production (*68*), prostaglandin E_2_ secretion (*69*), and low MHC-II expression, a phenotype generally associated with reduced T cell activation (*70*). Although the depleted KC niche can be restored through monocyte differentiation into KC-like cells (*30*, *37*), our transcriptome analyses in an intact niche highlight critical functional differences between moMF and KC. Despite moMF.2 showing transcriptional similarities to KC, they lacked expression of hallmark KC genes (*Clec4f*, *Marco*, *Timd4*, *Cd5l*, *Vsig4*, *Fcna*, and *Cdh5*) and maintained a proinflammatory profile, as indicated by the enriched allograft rejection pathway, which has been previously described for liver erythrophagocytes in a spherocytosis model (*67*). In addition, KC showed enrichment of xenobiotic metabolism pathways, reflected by increased expression of genes involved in heme/redox metabolism, lipid handling, and detoxification, indicating activation of metabolic programs linked to restorative and anti-inflammatory macrophage states. Combined with their immature state, as full maturation into bona fide KC–like cells typically requires 15 to 30 days (*71*), these characteristics suggest that moMF have limited potential to functionally replace resident KC in tolerance induction. These findings indicate a critical and nonredundant role for resident KC in pcRBC-mediated immune tolerance and reveal a previously undescribed immunomodulatory function of resident KC in EAE.

Building on the general tolerogenic capacity of KC and our findings identifying them as central mediators of pcRBC-induced tolerance, we hypothesized that the surviving KC undergo phenotypic transition into a restorative, anti-inflammatory state. As indicators, they up-regulated *Marco* and *Gpnmb* genes, while antigen presentation and interferon-stimulated genes were down-regulated. Notably, erythrophagocytosis has been shown to drive KC into an anti-inflammatory state characterized by *Marco* up-regulation, repression of MHC-II, and reduced antigen presentation capacity, resulting in the emergence of tolerogenic erythrophagocytes (*67*). In addition, *Marco* expression marks bona fide KC with homeostatic and tolerogenic properties in the human liver, supporting its association with noninflammatory macrophages (*31*). *Gpnmb* expression further supports this interpretation, as it is linked to M2-like polarization (*72*) and negative regulation of T cell activation (*73*). Consistent with this assumption, pathway analyses revealed decreased expression of genes involved in antigen processing, allograft rejection, inflammatory responses, and IFN-α/IFN-γ signaling at this time point, altogether indicating an anti-inflammatory transcriptional signature. In addition, it remains to be determined whether the reduced MHC-II profile reflects active transcriptional down-regulation in surviving KC or the selective loss of highly activated KC with elevated antigen presentation capacity. The overall decrease in MHC-II expression likely reflects both transcriptional silencing and compositional changes within the KC compartment that together promote the presentation of antigens in a tolerogenic way. At the protein level, we additionally observed increased PD-L1 expression in KC and moMF that have phagocytosed pcRBC, which is in line with similar observations previously reported for KC and LSECs that take up antigen-coupled nanoparticles (*69*), splenic macrophages that phagocytose apoptotic pcSplenocytes (*18*), or DCs in the liver and spleen after antigen-coupled nanoparticle injection (*51*). These findings suggest that PD-L1–mediated inhibitory signaling may be one component of the broader tolerogenic shift after treatment, which suggests a functional transition toward anti-inflammatory, tissue-repair functions consistent with the acquisition of tolerogenic properties.

Our findings point at a coordinated mechanism by which KC initiate a tolerance program upon pcRBC clearance. We observed a pronounced reduction in KC numbers after pcRBC treatment, attributable to cell death rather than migration to ldLN. Although surviving KC appear to be direct mediators of tolerance induction, it cannot be excluded that: (i) they require phagocytosis of apoptotic KC to obtain antigen in a tolerogenic fashion or (ii) that material from the apoptotic KC needs to be phagocytosed by other APCs to confer tolerance (*51*). Among such APC populations, LSECs are of particular interest given their well-established role in hepatic immune tolerance through antigen uptake and presentation (*74*, *75*). In a preliminary analysis, we found low levels of pcRBC membrane-derived material in LSECs 17 hours postinjection, whose relevance we will follow up on in the future. In addition, Casey *et al.* (*69*) proposed that LSECs contribute to and maintain nanoparticle-induced tolerance in the absence of KC. However, in our study, KC depletion completely abrogated pcRBC-mediated tolerance, hence suggesting that LSECs cannot compensate for the loss of KC in our setting. The antigen specificity of pcRBC-mediated EAE prevention in our model, which is consistent with prior work using peptide-coupled cells, suggests that the early tolerogenic intervention with disease-relevant antigen is sufficient to establish long-term antigen-specific immune tolerance despite decreased antigen presentation. We speculate that, given their self-renewal capacity (*76*), surviving KC likely resume proliferation after pcRBC clearance (*29*) and reestablish their niche. However, given their transient depletion and subsequent replacement, the extent to which the remodeled KC compartment contributes to the long-term maintenance of the tolerogenic state remains unclear. We therefore propose that KC primarily serve to initiate downstream tolerogenic processes. This initiation potentially involves downstream adaptive immune mechanisms such as induction of antigen-specific tolerogenic T cells, which could contribute to sustaining long-term immune tolerance independently of the original KC population.

We had initiated our study in the mouse model to provide additional information and mechanistic insight for translation in patients with MS, but we have to concede that our study has a number of limitations. First, external validity is limited because, at this stage, we used only one particular EAE model with one peptide for induction and a chronic nonrelapsing disease type; further data from other related progressive and relapsing-remitting models are warranted. On purpose, we restricted this study to the phagocyte compartment of the liver, leaving other important organs and cell types unaddressed at this moment. We could not yet identify the exact tolerogenic mechanism and the receptor mediating erythrophagocytosis. Last, repetitive treatment studies are needed to assess the long-term establishment of immune tolerance.

In conclusion, this study unequivocally establishes that resident KC are the orchestrators of pcRBC-mediated antigen-specific immune tolerance in EAE. Future studies should explore how KC interact with CD4^+^ T cells to induce antigen-specific immune tolerance. Understanding the key mechanisms of this process may foster further development of tolerogenic therapies to treat MS and other autoimmune diseases.

## MATERIALS AND METHODS

### Mice

WT (C57BL/6J) and transgenic mice used in this study are listed in table S1. All mice were bred in-house under specific and opportunistic pathogen–free conditions, were used for experiments in a conventional sterile animal facility at the Laboratory Animal Services Center of the University of Zurich in the absence of Federation of European Laboratory Animal Science Associations–listed pathogens, and were group housed in individually ventilated NexGen type II long cages (Allentown) with a maximum of five animals per cage. Cages were autoclaved with wood chips (LIGNOCEL Select, J. Rettenmaier & Söhne) as bedding material and a mouse house (red-transparent polycarbonate; ZOONLAB GmbH). Paper tissues (Uehlinger AG) and paper fibers (ARBOCEL Crinklets Natural, J. Rettenmaier & Söhne) were used as enrichment. The breeding food (3802 extrudate for specific and opportunistic pathogen–free and 3336 extrudate for sterile rooms; KLIBA NAFAG) was supplied ad libitum. The animals were kept under a controlled light cycle (14/10 hours), room temperature (RT; 21°C), and room humidity (45 to 60%). Health monitoring using sentinels on dirty cages was performed quarterly in accordance with Federation of European Laboratory Animal Science Associations guidelines. Personnel wore masks, bonnets, gloves, dedicated clothes, disposable overalls, and dedicated shoes. Animal experiments were performed under the following animal experimental licenses: ZH195/2018, ZH138/2022, ZH059/2022, ZH159/2025, ZH057/2026, and ZH004/2026.

All imported animals were allowed to acclimate for at least 2 weeks in our animal facility. Eight- to 14-week-old mice of both sexes were used in the experiments unless indicated otherwise. Stabilin 1 and 2 KO mice were 12 to 13 months old. For all pcRBC uptake experiments involving KO mice, age-matched WT control animals were included and analyzed in parallel, and statistical analyses were conducted within matched experimental blocks, as described in the corresponding figure. Mice were randomly assigned to experimental groups. The experimenters remained blinded to treatment allocation throughout the clinical scoring of EAE. All breeding and experimental procedures were conducted in accordance with national, federal, and institutional regulations in Switzerland and Germany. Sample size for exploratory experiments was set at least six or more animals. The actual number of animals is indicated in the figure legends.

### RBCs coupling

C57BL/6-Mavs^<em1Thbu>^ (MAVS KO), C57BL/6-Sting1^<em1ThBu>^ (STING KO), C57BL/6-*Ticam2*^<em1ThBu>^ (TRAM KO), B6;129S1-*Tlr3*^<tm1Flv>^/J (TLR3 KO), and B6.129P2-*Tlr4*^<tm1Aki>^ (TLR4 KO) mice were used as donor mice for the collection of blood and the preparation of pcRBC. The suitability of RBCs from gene-deficient mice compared to RBCs from WT mice has been confirmed in our laboratory. In detail, mice were euthanized with CO_2,_ and blood was collected through heart puncture in micro-citrate tubes using 25-gauge needles. The blood was filtered through a 40-μm cell strainer and transferred to a polystyrene tube. The sample was centrifuged at 350*g* (10 min at RT), and the plasma layer was discarded following centrifugation. The remaining sample was washed in 15 ml of 0.9% saline at 290*g* [5 acceleration (acc), 4 deceleration (dec)] for 10 min at RT. RBCs were quantified using a Neubauer chamber. The coupling reaction was performed by first pulsing the RBCs (3 × 10^9^ cells/ml) with MOG_35–55_ or OVA_323–339_ (0.924 mg/ml; hi: 0.924 mg/ml, med: 0.462 mg/ml, and low: 0.132 mg/ml for the peptide dose experiment) and subsequently mixing the pulsed cell suspension with the cross-linker EDC (3 mg/ml). The cells were incubated for 1 hour under mild shaking and were subsequently washed twice as above and counted for further applications.

### RBCs labeling

RBCs labeling was performed using Pkh26 Red Fluorescent Cell Linker Kit for General Cell Membrane Labeling (#MINI26-1KT, Sigma-Aldrich) or CTFR Cell Proliferation Kit (#C34572, Invitrogen). For pkh26, 10 × 10^9^ RBCs were resuspended in 1 ml of Diluent C and were mixed with 1 ml Diluent C containing 4 μM pkh26. The staining reaction was carried out for 5 min at RT in the dark and was stopped by addition of 10 ml of RPMI 1640 medium containing 10% fetal bovine serum (FBS). Cells were washed twice with 0.9% saline by centrifugation at 290*g* (5 acc and 4 dec for 10 min at RT). After counting, the RBCs were processed for peptide coupling as described above and were immediately injected into mice. RBCs labeling was alternatively performed with CTFR Cell Proliferation Kit (#C34572, Invitrogen) according to the manufacturer’s instructions. Briefly, RBCs were first coupled to peptide as described above and later labeled with CTFR. In detail, up to 10 × 10^9^ RBC were stained in 1 ml of 0.9% saline containing 5 μM CTFR (1 μl) for 20 min at RT under occasional mild mixing. The staining reaction was stopped using 10 ml of RPMI 1640 medium containing 10% FBS. The RBCs were spun down and subsequently washed once in 0.9% saline. The RBCs were counted before injection into mice. In all experiments, the efficiency of RBC labeling was assessed by flow cytometry.

### pcRBC injections

C57Bl/6J and transgenic mice were injected intravenously with 2 × 10^9^ dye-labeled or unlabeled pcRBC or 0.9% saline (control) and were euthanized 2 or 17 hours postinjection. Livers and spleens were processed for single-cell suspensions as described below. Myeloid cell subsets and the uptake of pcRBC were assessed by flow cytometry. In KO and DTx-treated animals, pcRBC uptake was assessed 2 hours post–pcRBC injection, with WT or saline-treated animals, respectively, serving as controls.

### Sample processing for multidimensional fluorescence microscopy

After euthanizing the mice with CO_2_, livers were perfused first with phosphate-buffered saline (PBS) until fully discolored, followed by ∼30 ml of cold 4% paraformaldehyde (PFA) using a peristaltic pump (P70, Instech). After overnight incubation in cold 4% PFA, the samples were dehydrated in 10% sucrose overnight and 30% sucrose for 24 to 72 hours. Next, the samples were frozen on dry ice or embedded in optimal cutting temperature (#81-0771-00, CellPath) cryoblocks and stored in −80°C. Each cryoblock was serially sectioned on Leica CM1950 (Leica Biosystems GmbH), and 6-μm sections were placed on SuperFrost Ultra Plus glass slides. For the immunofluorescence staining, after one wash with PBS, slides were incubated with 1% bovine serum albumin and 0.1% of Triton X-100 in PBS at RT for 40 min. After blocking, samples were loaded on COMET (Lunaphore) and were stained with primary and corresponding secondary antibodies in Multistaining Buffer (BU06, Lunaphore Technologies) according to a fully automated protocol of sequential immunofluorescence. For each cycle, staining for two antibodies, imaging, and elution were performed, and the data were analyzed on the Horizon software. Imaging was performed at a ×20 magnification with equipment maintained by the Center for Microscopy and Image Analysis, University of Zurich. The antibodies used in this study are presented in table S2.

### EAE induction

Mice were immunized by subcutaneous injection of 200 μl of induction solution, containing 200 μg of MOG_35–55_ (#EP02030_5, Peptides & Elephants) emulsified in CFA (#231131, BD), with an additional 500 ng of *Mycobacterium tuberculosis* (#231141, BD) (100 μl of emulsion mix per flank). On days 0 and 2 postimmunization, mice were intraperitoneally injected with 666 ng of PTX (#179A, LubioScience) in 100 μl of PBS. Starting from day 5, weight and clinical symptoms of EAE were monitored daily using the following criteria: 0, no clinical signs; 0.5, distal limp tail; 1, complete limp tail; 1.5, limp tail and hind limb paralysis; 2, unilateral partial hind limb paralysis; 2.5, bilateral partial hind limb paralysis; 3, complete bilateral hind limb paralysis.; 3.5, complete bilateral hind limb and partial forelimb paralysis; 4, moribund (mouse completely paralyzed); 5, dead. Exclusion criteria regarding weight loss, disease severity, and overall health status were applied at all times according to our animal application.

In detail, exclusion criteria were met if a weight loss of 25% or more, compared to day 0 postimmunization, was observed. Furthermore, euthanasia was performed if a score of ≥3 persisted for more than 7 days, if a score of 3.5 persisted for more than 3 days, or if a score of 4 was recorded. When a mouse died during the experiment or met exclusion criteria, it was consistently assigned a score of 5 until the end of the experiment.

### EAE induction and pcRBC treatments

For the antigen-specificity experiment, female mice were injected with 1 × 10^9^, 1.5 × 10^9^, or 2 × 10^9^ pcRBC coupled to MOG_35–55_ or OVA_323–339_ (0.924 mg/ml) or PBS/saline as control. MOG_35–55_– or OVA_323–339_–injected mice were pooled under pcRBC and pcRBC(OVA) groups, respectively. All three groups [pcRBC, pcRBC(OVA), and PBS/saline] were tested in three independent experiments. In addition, the pcRBC and PBS/saline groups were included in three further independent experiments, resulting in a total of six experiments for these two groups.

For the prophylactic cell-dose experiment, female mice were injected with 1 × 10^9^, 1.5 × 10^9^, or 2 × 10^9^ pcRBC coupled to MOG_35–55_ (0.924 mg/ml) or PBS/saline as control. The 1 × 10^9^ and 1.5 × 10^9^ groups were pooled and thereafter referred to as ≤1.5 × 10^9^ group. The PBS/saline control group was identical to the control group of the antigen-specificity experiment. All three groups (≤1.5 × 10^9^ cells, 2 × 10^9^ cells, and PBS/saline) were included in one independent experiment, with additional independent experiments performed for the lower-dose/PBS and higher-dose/PBS groups, resulting in a total of six experiments.

For the prophylactic peptide-dose experiment, female mice were injected with 2 × 10^9^ pcRBC coupled to different concentrations of MOG_35–55_ as follows: hi MOG (0.924 mg/ml), med MOG (0.462 mg/ml), low MOG (0.132 mg/ml), or 0.9% saline as control. Seven days after pcRBC or pcRBC(OVA) injection, they were induced with EAE. Following EAE induction as above, mice were monitored for signs of paralysis for 28 days.

For the therapeutic treatments, female mice were induced with EAE as above on day 0. For the disease onset experiment, once all mice exhibited the first symptoms of disease induction (score: 0.5, distal limp tail), they were intravenously injected with 2 × 10^9^ pcRBC or pcRBC(OVA) (control) coupled to the highest dose of peptide (0.924 mg/ml). Similarly, for the later-stage treatment, once at least one mouse in each group [pcRBC(OVA) or pcRBC] showed a score of 1.5 (limp tail and hind limb paralysis), mice were injected as described above for the disease-onset treatment. In both experiments, mice were monitored for signs of paralysis for 28 days.

### Experiments with KC-depleted animals

To deplete KC, KC-DTR mice were injected intraperitoneally with 100 ng of DTx (#322326, Sigma-Aldrich) in 0.9% saline or 0.9% saline (control) and were assessed for the absence of KC 12 hours, 7 days, and 14 days later. The percentage of CLEC4F^+^ (tdT^+^) cells was evaluated by flow cytometry.

For the pcRBC uptake experiments, KC-DTR mice were intraperitoneally treated with 100 ng of DTx (or 0.9% saline) for the depletion of KC and, 12 hours later, were injected intravenously with 2 × 10^9^ CTFR-prelabeled pcRBC or 0.9% saline; mice were euthanized 2 hours posttreatment. The uptake of CTFR-pcRBC by liver NPC and the percentage of remaining CTFR-pcRBC in the circulation were assessed by flow cytometry. In this case, the blood was collected from the inferior vena cava at the beginning of liver perfusion.

For the prophylactic EAE experiment post–KC depletion, WT animals were used as controls for both disease induction and tolerization and were subjected to the same procedures as KC-DTR mice. For this, both strains were intravenously injected with DTx and, 12 hours later, were injected with pcRBC or 0.9% saline. Seven days post–pcRBC treatment, the mice were induced with EAE as described above and monitored for clinical signs of paralysis for 28 days.

### Histological analysis

Animals were perfused with PBS, followed by 4% PFA. After fixation, whole heads and vertebrae were sectioned and dehydrated for paraffin embedding. FFPE-embedded tissues were cut at a thickness of 3 μm and rehydrated. Histological stainings for H&E, Luxol Fast Blue, and Periodic Acid Schiff were performed as described by Locatelli *et al.* (*77*).

### Preparation of single-cell suspensions

#### Liver digestion and liver NPC preparation

After euthanizing the mice with CO_2,_ the liver was digested for the preparation of NPC, according to Pfefferlé *et al.* (*67*). In detail, using a peristaltic pump, the liver was perfused with warm Hanks’ balanced salt solution (HBSS) (#14175-095, Gibco) containing 2 mM EDTA (#15575-038, Invitrogen) at 37°C until fully discolored and was subsequently digested with warm HBSS-MgCl_2_/CaCl_2_ buffer (#14025-050, Gibco) containing 0.05% Collagenase D (#11088882021, Sigma-Aldrich) (37°C). Once fully digested, the Glisson’s capsule was removed, and the liver was mechanically dissociated and homogenized in additional ice-cold PBS in a petri dish using a 10-ml serological pipette. The resulting cell suspension was filtered through a 100-μm cell strainer and centrifuged twice at 100*g* (3 min at 4°C) for hepatocyte removal (pellet). The resulting cell suspension (supernatant) was centrifuged at 300*g* (5 min at 4°C) to obtain a cell pellet. RBCs were lysed using RBC lysis buffer (#420302, BioLegend) for 3 min at RT. Lysis was stopped by adding cold PBS, and the cell suspension was centrifuged at 350*g* (5 min at 4°C). Following centrifugation, the supernatant was discarded, and the cell pellet (NPC) was resuspended in PBS, counted, and stored at 4°C until further processing.

#### Splenocyte preparation

Following euthanasia with CO_2_, the spleen was collected and mashed with a syringe plunger through a 70-μm nylon mesh cell filter. The filter was washed with PBS, and the obtained cell suspension was spun down at 350*g* (5 min at 4°C). After centrifugation, the supernatant was discarded, and the cell pellet was resuspended in 1 ml of RBC lysis buffer for 5 min at RT. Lysis was stopped by adding 10 ml of PBS, and the resulting cell suspension was further centrifuged at 350*g* (5 min at 4°C). Following centrifugation, the supernatant was discarded, and the cell pellet was resuspended in PBS, counted, and stored at 4°C until further processing.

#### LN cell preparation

Following euthanasia with CO_2_ and after spleen collection, hepatic LN were collected. After liver digestion, inguinal LN were also collected. LNs were cut into small pieces with scissors and were digested in a six-well plate in 3 ml of HBSS-MgCl_2_/CaCl_2_ buffer (#14025-050, Gibco), containing 10% FBS, Collagenase D (0.4 mg/ml; #11088882021, Sigma-Aldrich), and DNase I (0.1 mg/ml; #10104159001, Sigma-Aldrich) for 30 min at 37°C. After incubation, the digested tissue was homogenized with a 22-gauge syringe. The homogenate was filtered through a 70-μm strainer, topped up to 10 ml with PBS, and centrifuged at 1500 rpm (10 min at 4°C). The pellet was resuspended in PBS, counted, and stored at 4°C until further processing.

### Antibody staining

#### Surface staining

Cells were plated in PBS in a U-bottom 96-well plate (#82.1582, SARSTEDT AF) and spun down (350*g* for 5 min at 4°C). Following centrifugation, the cell pellet was resuspended in 100 μl of Zombie NIR Fixable Viability Kit (1:1000 in PBS; #423102, BioLegend) and stained for 20 min (4°C in the dark). After topping up with PBS, the cells were washed, and the supernatant was discarded. The cell pellet was stained with TruStain FcX anti-mouse CD16/32 antibody (#101319, BioLegend) for 10 min (4°C in the dark), prepared at 1:50 dilution in fluorescence-activated cell sorting (FACS) buffer [PBS containing 10% FBS and 2 mM EDTA (#15575-038, Invitrogen)]. Without washing, the antibody mix prepared in FACS buffer was added to the respective wells, and cells were stained for 20 min (4°C in the dark).

#### Intracellular TER119 staining

Following surface staining, cells were fixed and permeabilized with PFA and saponin buffer (eBioscience Foxp3/Transcription Factor Staining Buffer Set, #00-5523-00, Thermo Fisher Scientific) for 45 min at RT. Following fixation and permeabilization, the cells were intracellularly stained for TER119-PE (TER119, #100-0451, StemCell) prepared at a 1:100 dilution in 1× Permeabilization Buffer for 60 min at RT. To remove unbound antibodies, stained cells were washed twice with 1× Permeabilization Buffer (350*g* for 5 min at 4°C) and resuspended in FACS buffer for acquisition.

#### Antibody panels

Liver NPC were stained with the following surface markers: CD45-PerCP-Cy5.5 (clone 30-F11, #103131, BioLegend), CD80-PE (clone 16-10A1, #104708, BioLegend), PD-L1-PE-Dazzle594 (clone 10F.9G2, #124324, BioLegend), I-A/I-E (MHC-II) -AF700 (clone M5/114.15.2, #107621, BioLegend), TIM4-PE-Cy7 (clone RMT4-54, M5/114.15.2, #130009, BioLegend), F4/80-BV421 (clone BM8, #123131, BioLegend), CD11b-BV605 (clone M1/70, #101237, BioLegend), CD11c-BV785 (clone N418, #117335, BioLegend), and Ly6C-FITC (clone HK1.4, #128005, BioLegend), and Ly6G/Ly6C-APC (clone RB6-8C5, #108412, BioLegend). Pkh26- and CTFR-labeled pcRBC uptake was assessed in the phycoerythrin (PE) and APC fluorescence channels, respectively. All surface staining antibodies were used in a 1:400 dilution in FACS buffer. Liver KC were identified as single live CD45^+^F4/80^hi^CD11b^+^ cells, monocytes as single live CD45^+^F4/80^−^CD11b^+^Ly6C^+^Ly6G^−^ cells, neutrophils as CD45^+^F4/80^−^CD11b^+^Ly6C^+^Ly6G^+^ cells, and moMF as live CD45^+^F4/80^+^CD11b^+^ cells. The uptake of pcRBC was determined by the percentage of dye^+^CD11b^+^ cells.

### Flow cytometry data acquisition and data analysis

Samples were acquired on an LSR II Fortessa 4L at the Cytometry Facility, University of Zurich, and were analyzed using FlowJo version 10.10.0 (Tree Star) software.

### Cell preparation for scRNA-seq analysis

WT mice were intravenously injected with 2 × 10^9^ pcRBC or saline and were euthanized 2 and 17 hours later. Livers were processed as above to prepare single-cell suspensions. Liver NPC were enriched for CD45^+^ cells using CD45 (TIL) MicroBeads (#130-110-618, Miltenyi), and a small fraction of these was controlled with flow cytometry to confirm the enrichment efficiency. Splenocytes and liver CD45^+^ NPC were counted and fixed according to the Chromium Fixed RNA Profiling for Multiplexed Samples kit (#1000414, 1000496, and 1000422, 10x Genomics). After fixation, samples were hybridized to probe sets and pooled for multiplexing (liver and spleen separately). Next, after cell partition into gel bead-in-emulsions, ligation, and extension reactions, the 10x barcoded library was constructed. QC and sequencing of the library were performed by the Functional Genomics Center Zurich (FGCZ). Sequencing was conducted on Illumina NovaSeq X Plus, with a sequencing depth of ∼7000 to 11,000 reads per cell. The experiment was performed in two rounds as follows: *n* = 1 (saline; 2 hours), *n* = 1 (pcRBC; 2 hours), *n* = 1 (saline; 17 hours), and *n* = 1 (pcRBC; 17 hours), resulting in two samples per condition in total.

### Analysis of scRNA-seq data

#### Processing and QC

Cell Ranger 8.0.0 was used by the FGCZ to demultiplex FASTQ reads, align reads to the mouse reference genome (GRCm39, release M31), and generate the UMI count matrices for each condition with the v1.0.1 (mm10) probe set. To eliminate ambient RNA, we ran the remove-background command of CellBender 0.3.0 (*78*) with default settings on the unfiltered (raw) .h5 file obtained with Cell Ranger. Following, we filtered doublets using scDblFinder 1.14.0 (*79*) and removed cells lying more than three median absolute deviations away from the sample mean in at least one of the following metrics: total UMI count, number of detected genes (with UMI > 1), and fraction of mitochondrial reads.

#### Integration and clustering

After QC, each matrix was loaded with Seurat 5.1.0 (*80*) and normalized and variance stabilized using SCTransform, regressing out the fraction of mitochondrial reads with the vars.to.regress parameter. Following, the SCT models were prepared for integration using the PrepSCTFindMarkers function. Then, we integrated the samples using IntegrateLayers with the Harmony method and computed the dimensionality reduction (UMAP). We used FindNeighbors and FindClusters with a resolution of 0.25 and the Leiden algorithm to cluster the data. The smallest of the resulting clusters, cluster 13, was removed because it belonged only to the second pcRBC sample at 17 hours.

We annotated clusters manually using known markers and literature (*28*, *62*, *64*). Following, myeloid clusters were extracted to be separately analyzed. We reclustered this data with a resolution of 0.15 to obtain a separate clustering of myeloid cells. Then, we subclustered cluster 3, which was composed of different populations, using the FindSubCluster function with resolution of 0.15, resulting in three additional clusters (later identified as moMF.1, monoLy6c^+^, and monoLy6c^–^). The clusters were manually annotated with known markers. To ensure the correct identification of KC, we defined the KC cluster to be composed of only Clec4f-expressing cells. This revealed a population of cells adjacent to the KC cluster, exclusive to the pcRBC condition at 17 hours, which we labeled as moMF.2.

#### Differential gene expression

Differentially expressed genes (DEGs) were identified using the FindMarkers function of Seurat including genes with a minimum expression of 15% and using the MAST algorithm with the experiment as latent variable. We consider DEGs to be significant and relevant with a false discovery rate (FDR) < 0.01 and |logFC| ≥ 1.5.

#### Gene set enrichment analysis

We first performed differential gene expression analysis using FindMarkers without filtering for expression percentage, logFC, or significance. Results were ranked by the logarithm of the *P* value multiplied by the fold change sign and tested for enrichment using fgsea 1.26 with gene sets obtained from MSigDB version 2025.1.Mm. The genes of the KEGG_Ferroptosis pathway were also used as a gene set. Enrichment was considered significant with FDR < 0.05.

### Statistical and other analyses

Statistical analyses and graphical representations were performed on GraphPad Prism v10. For comparisons between two groups, either a parametric *t* test or a nonparametric Mann-Whitney test was used, depending on data distribution. For comparisons involving more than two groups, a one-way analysis of variance (ANOVA) with multiple comparisons depending on the experimental question was used. In cases of nonequal SDs, the Brown-Forsythe ANOVA test was applied. Statistical significance was defined as *P* < 0.05.

#### EAE experiments

For the EAE experiments, the mean AUC of the clinical score for each group was calculated after obtaining the AUC of each individually plotted mouse on GraphPad Prism, setting the baseline at *Y* = 0 and ignoring peaks that are less than 10% of the distance from the minimum to the maximum *Y* (default). AUC of each mouse was then plotted as mean AUC ± SEM per group and tested for statistically significant difference using a parametric *t* test, nonparametric Mann-Whitney test, a one-way ANOVA, or a Brown-Forsythe ANOVA, depending on the number of groups and variance distribution. The mean score represents the average of clinical scores of each individual mouse and was demonstrated as mean of mean clinical scores ± SEM per group and tested for statistically significant difference using a parametric *t* test or nonparametric Mann-Whitney test as indicated in the respective figure legend. Specifically for the antigen specificity and cell-dose analyses, EAE severity was summarized as the trapezoidal AUC and normalized to the duration of the corresponding experiment. For the antigen specificity experiment, the normalized AUC was analyzed using an incomplete-block linear model including treatment group and experiment as fixed effects. For the cell-dose analysis, each dose was compared with PBS using experiments containing both that dose and PBS, using linear models with treatment and experiment block as fixed effects. In the experiment in which both doses were tested in parallel, the two doses and PBS were analyzed by one-way ANOVA, followed by pairwise comparisons. *P* values from the pairwise comparisons were adjusted using the Benjamini-Hochberg method (*P* < 0.05).

For the incidence and onset-peak disease analysis, disease incidence was calculated as the *N* of mice that developed symptoms (score: 0.5) divided by the total *N* of mice per group. The mean onset and peak disease day ± SD and the mean of maximum (peak) score of each mouse were calculated and depicted in figs. S1 (C, G, and K), S2C, S3C, and S16B for the respective EAE experiments. Mice that remained symptom-free mice (score: 0) were calculated in the incidence as nonresponders. These mice were excluded from onset-peak disease analysis but were included in the AUC and clinical score analysis. Mice that died spontaneously were excluded from the analysis. For mice that had a score of 0.5 throughout the course of disease, the onset day was considered equal to the peak day.
