# Supplementary material for: Kupffer cells are key mediators of antigen-specific immune tolerance by peptide-coupled red blood cells
Source: Sci Adv. 2026 Oct 7;12(41):eaeg6770. doi: 10.1126/sciadv.aeg6770 (PMC13644765; doi:10.1126/sciadv.aeg6770)
Supplement: Supplementary file 1 [file sciadv.aeg6770_sm.pdf]

Science Advances
AAAS

Supplementary Materials for

Kupffer cells are key mediators of antigen-specific immune tolerance by peptide-coupled red blood cells

Vasileia Kalaitzaki *et al.*

Corresponding author: Andreas Lutterotti, alutterotti@cellerys.com; Thorsten Buch, thorsten.buch@uzh.ch

Sci. Adv. **12**, eaeg6770 (2026)
DOI: 10.1126/sciadv.aeg6770

The PDF file includes:

Figs. S1 to S16
Tables S1 and S2
Legends for tables S3 to S13

Other Supplementary Material for this manuscript includes the following:

Tables S3 to S13

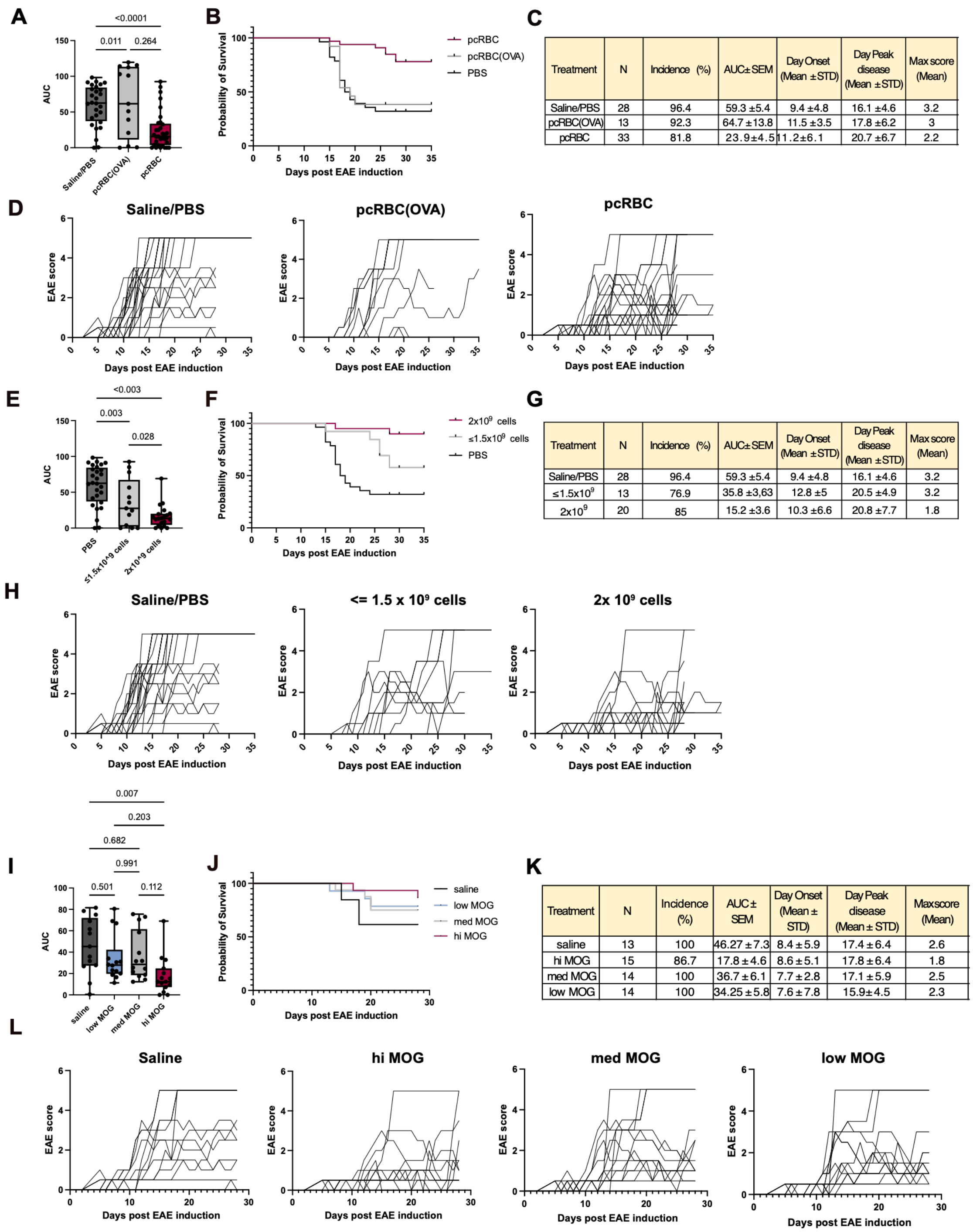

C

Treatment	N	Incidence (%)	AUC± SEM	Day Onset (Mean ±STD)	Day Peak disease (Mean ±STD)	Max score (Mean)
Saline/PBS	28	96.4	59.3 ±5.4	9.4 ±4.8	16.1 ±4.6	3.2
pcRBC(OVA)	13	92.3	64.7 ±13.8	11.5 ±3.5	17.8 ±6.2	3
pcRBC	33	81.8	23.9±4.5	11.2±6.1	20.7 ±6.7	2.2

G

Treatment	N	Incidence (%)	AUC± SEM	Day Onset (Mean ±STD)	Day Peak disease (Mean ±STD)	Max score (Mean)
Saline/PBS	28	96.4	59.3 ±5.4	9.4 ±4.8	16.1 ±4.6	3.2
≤1.5x10^9	13	76.9	35.8 ±3,63	12.8 ±5	20.5 ±4.9	3.2
2x10^9	20	85	15.2 ±3.6	10.3 ±6.6	20.8 ±7.7	1.8

K

Treatment	N	Incidence (%)	AUC± SEM	Day Onset (Mean ± STD)	Day Peak disease (Mean ± STD)	Maxscore (Mean)
saline	13	100	46.27 ±7.3	8.4 ± 5.9	17.4 ± 6.4	2.6
hi MOG	15	86.7	17.8 ± 4.6	8.6 ± 5.1	17.8 ± 6.4	1.8
med MOG	14	100	36.7 ± 6.1	7.7 ± 2.8	17.1 ± 5.9	2.5
low MOG	14	100	34.25 ± 5.8	7.6 ± 7.8	15.9±4.5	2.3

Fig. S1. Prophylactic pcRBC tolerization is antigen-specific, cell- and peptide-dose dependent. (A-D) *Antigen specificity experiment.* **(E-H)** *Cell-dose titration.* **(I-L)** *Peptide-dose titration.* **(A, E, I)** Area under the curve (AUC) of clinical scores. **(B, F, J)** Kaplan-Meier survival curves. **(C, G, K)** Number of mice (N) per group, Incidence of disease, AUC, Day of Onset, Day of peak disease, and Maximum score per group. **(D, H, L)** Spaghetti plots of disease development

of individual mice per group. Statistical analysis: **(A)** Duration-normalized AUC was analyzed using an incomplete-block linear model with treatment group and experiment as fixed effects. **(E)** The duration-normalized AUC for each dose was compared with experiment-matched PBS controls using linear models with treatment and experiment as fixed effects. The direct dose comparison was restricted to the experiment in which both doses were tested in parallel. **(A, E)** Pairwise p-values were adjusted using the Benjamini-Hochberg method. **(I)** One-way ANOVA with Tukey's post-hoc multiple comparisons test. Statistical significance: $p < 0.05$.

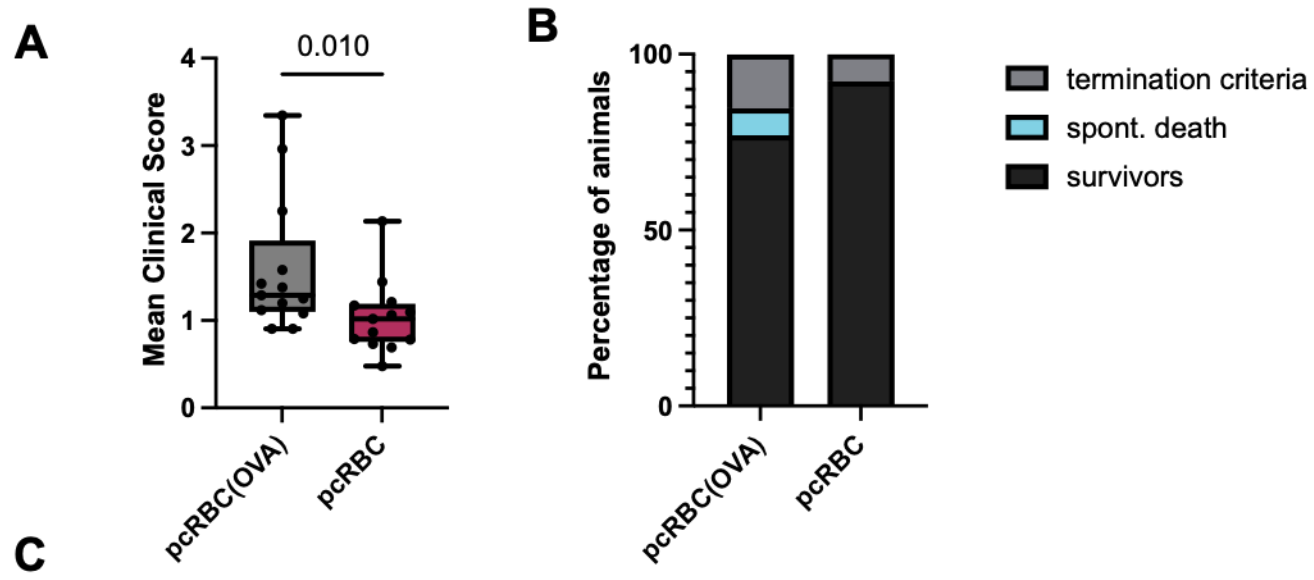

C

Treatment	N	Incidence (%)	AUC ± SEM	Day Onset (Mean ± STD)	Day Peak disease (Mean ± STD)	Max score (Mean)
pcRBC(OVA)	13	100	40.6 ± 3.9	7.1 ± 1.8	17.8 ± 4.4	3
pcRBC	13	100	26.3 ± 2.6	7.3 ± 1.7	20 ± 2.6	2.5

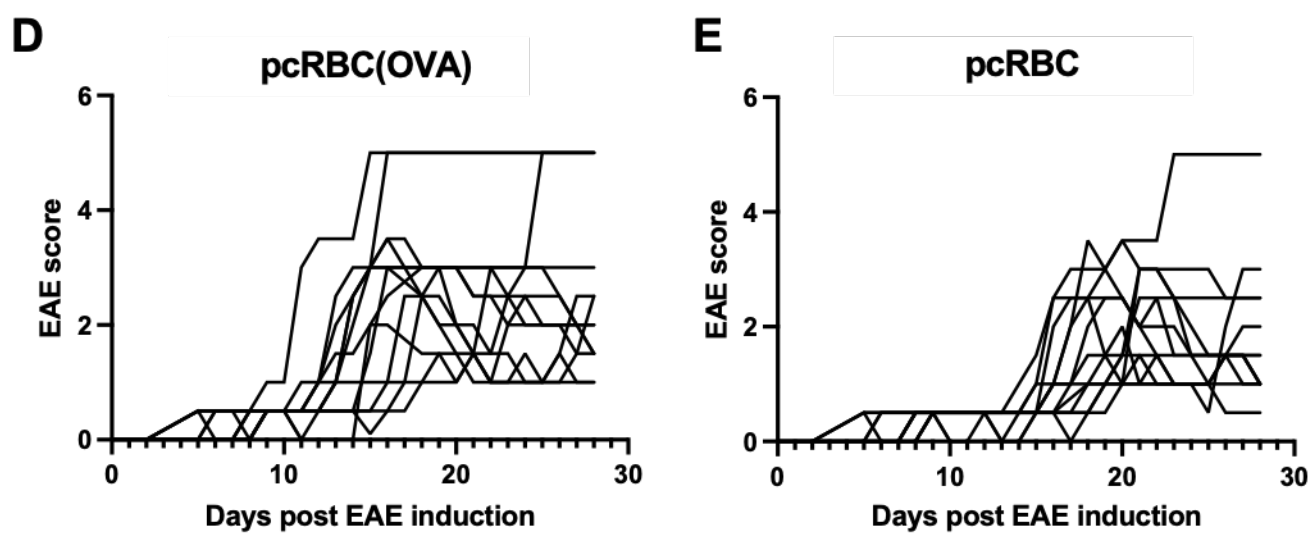

Fig. S2. Therapeutic pcRBC treatment ameliorates EAE. (A) Mean clinical scores of OVA-RBC and MOG-RBC groups. **(B)** Percentage of animals that survived, died spontaneously, or reached withdrawal criteria. **(C)** Number of mice (N) per group, area under the curve (AUC), Incidence of disease, Day of Onset, Day of peak disease, and Maximum score per group. **(D, E)** Spaghetti plots of disease development of individual mice per group. Data representative of 3 independent experiments (n=13/group). Non-parametric Mann-Whitney t-test was performed for the Mean scores between OVA-RBC and MOG-RBC groups. Statistical significance: $p < 0.05$.

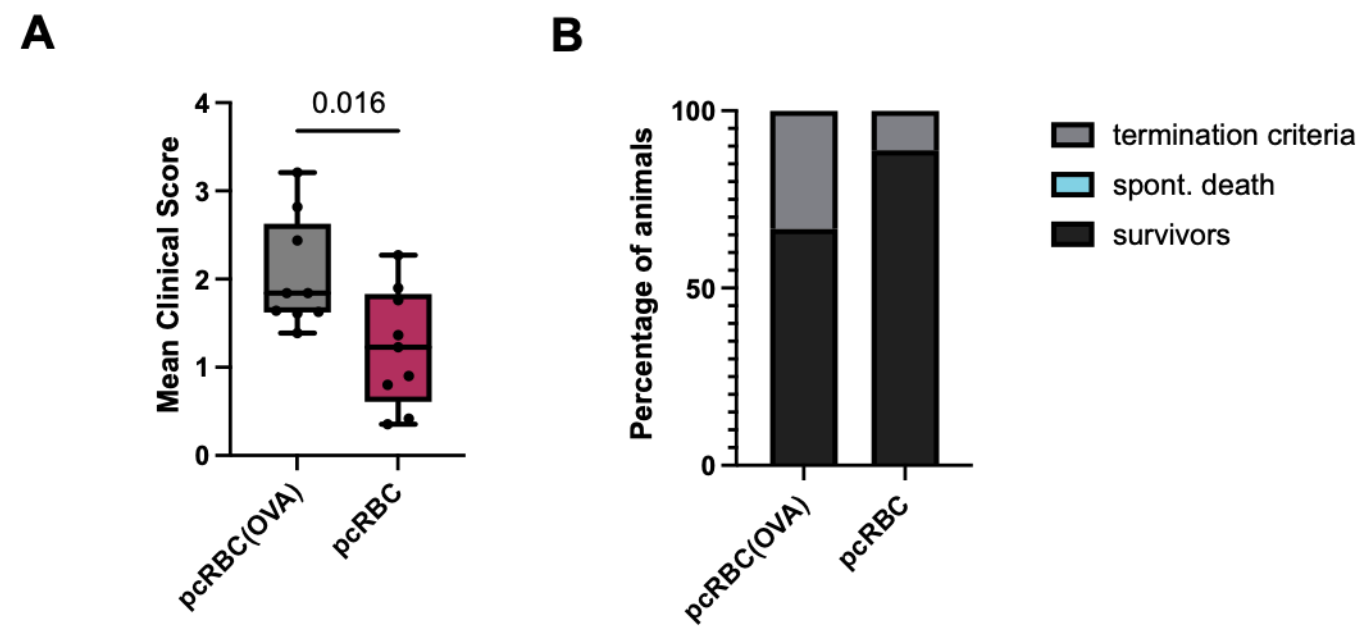

C

Treatment	N	Incidence (%)	AUC± SEM	Day Onset (Mean ± STD)	Day Peak disease (Mean ± STD)	Max score (Mean)
pcRBC(OVA)	9	100.0	49.3 ±4.9	8.8 ±1.6	17.4 ±2.4	3.2
pcRBC	9	100.0	29.5 ±5.3	8.6 ±1.4	15.7 ±3.8	2.3

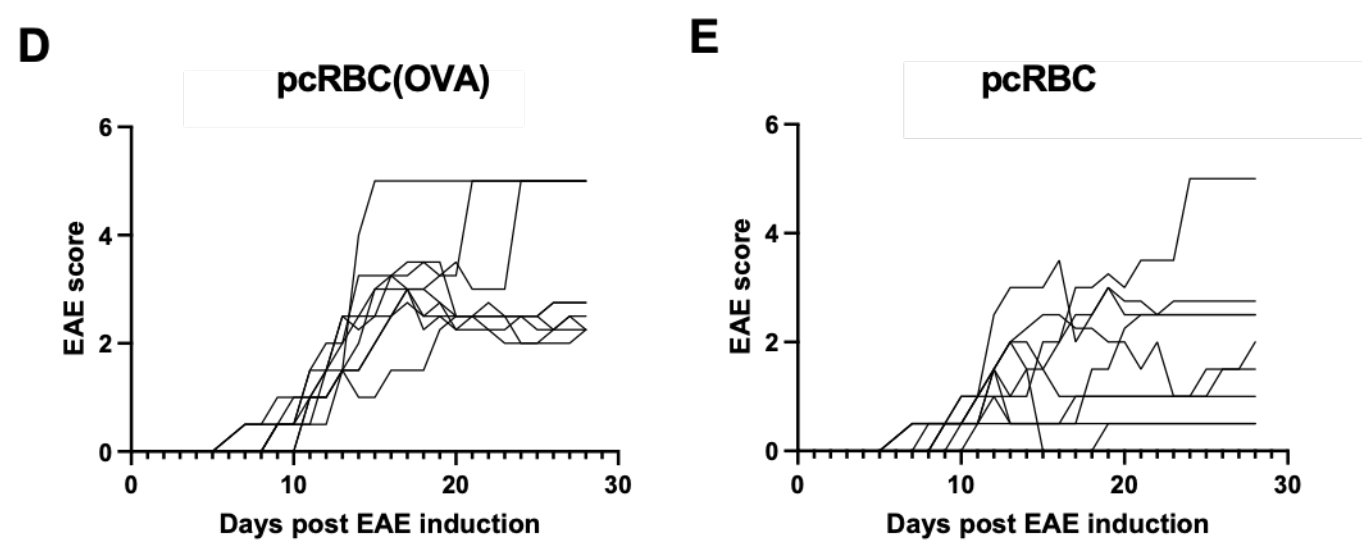

Fig. S3. Therapeutic pcRBC treatment during established disease ameliorates EAE. (A) Mean clinical scores of OVA-RBC and MOG-RBC groups. **(B)** Percentage of animals that survived, died spontaneously, or reached withdrawal criteria. **(C)** Number of mice (N) per group, area under the curve (AUC), Incidence of disease, Day of onset, Day of peak disease, and maximum score per group. **(D, E)** Spaghetti plots of disease development of individual mice per group. Data representative of 2 independent experiments (n=9/group). Unpaired Welch's t-test was performed for the Mean scores between OVA-RBC and MOG-RBC groups. Statistical significance: $p < 0.05$.

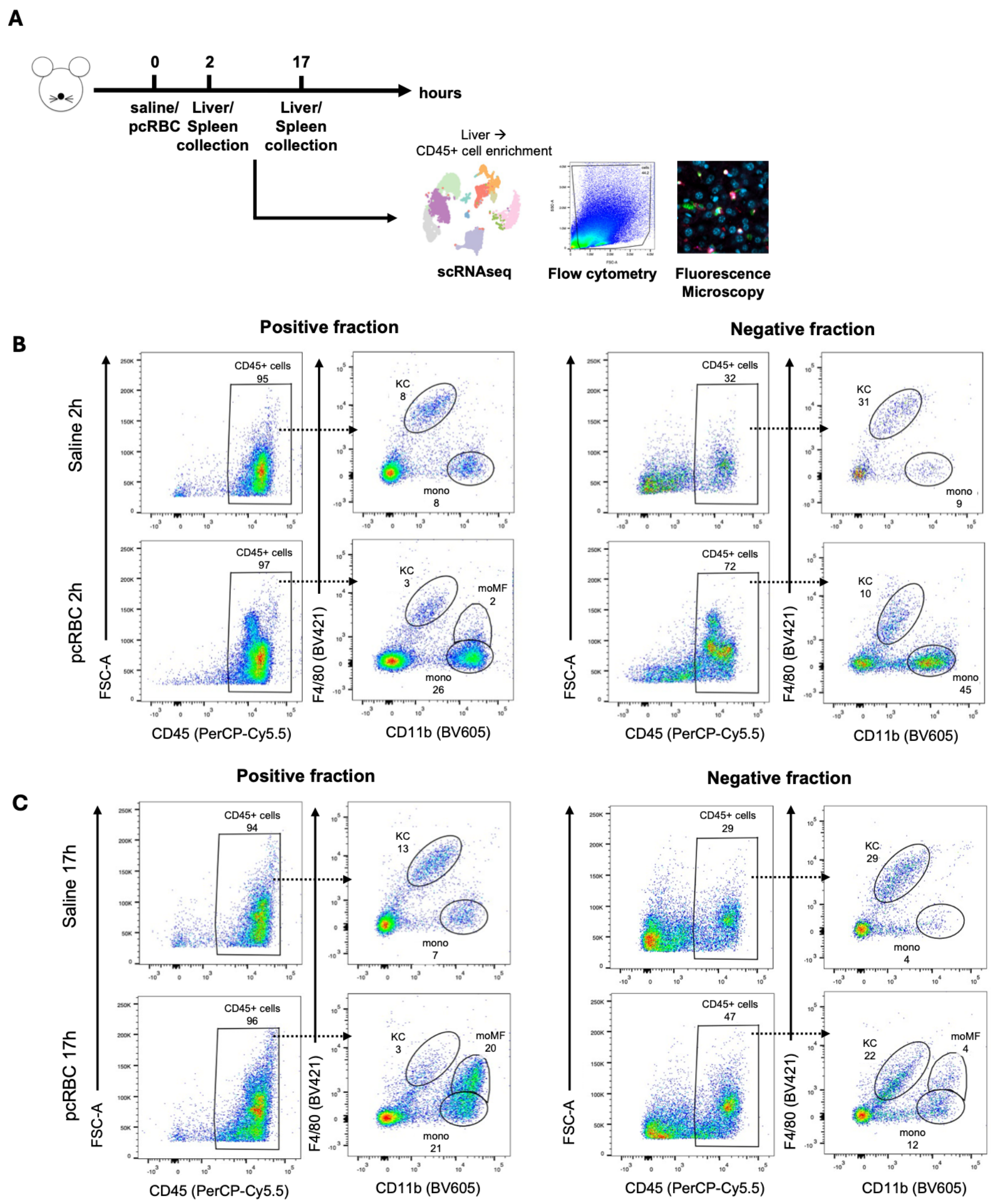

Fig. S4. Experimental plan and CD45⁺ purity control of scRNAseq input data (first run). (A) WT C57BL/6J mice were i.v. injected with 2×10^9 pcRBC coupled with 0.9 mg/ml MOG35-55 or 0.9% saline as control and were euthanized 2 h or 17 h later. After *in situ* digestion of the liver, single-cell suspensions were prepared for single-cell RNA sequencing (scRNAseq) or flow cytometry analysis. For scRNAseq analysis, liver NPC were enriched for $CD45^+$ cells and then processed for library preparation and sequencing (see Materials and Methods, *Cell preparation for scRNAseq analysis*). For microscopy analysis, livers were perfused and fixed as described in Materials and Methods (*Sample processing for multi-dimensional fluorescence microscopy*). Quality control of positive (left) and negative (right) fractions of liver NPC post $CD45^+$ enrichment of saline 2 h and pcRBC 2 h samples **(B)** and saline 17 h and pcRBC 17 h samples **(C)**. The cells are gated as FSC-SSC single live $CD45^+$ cells.

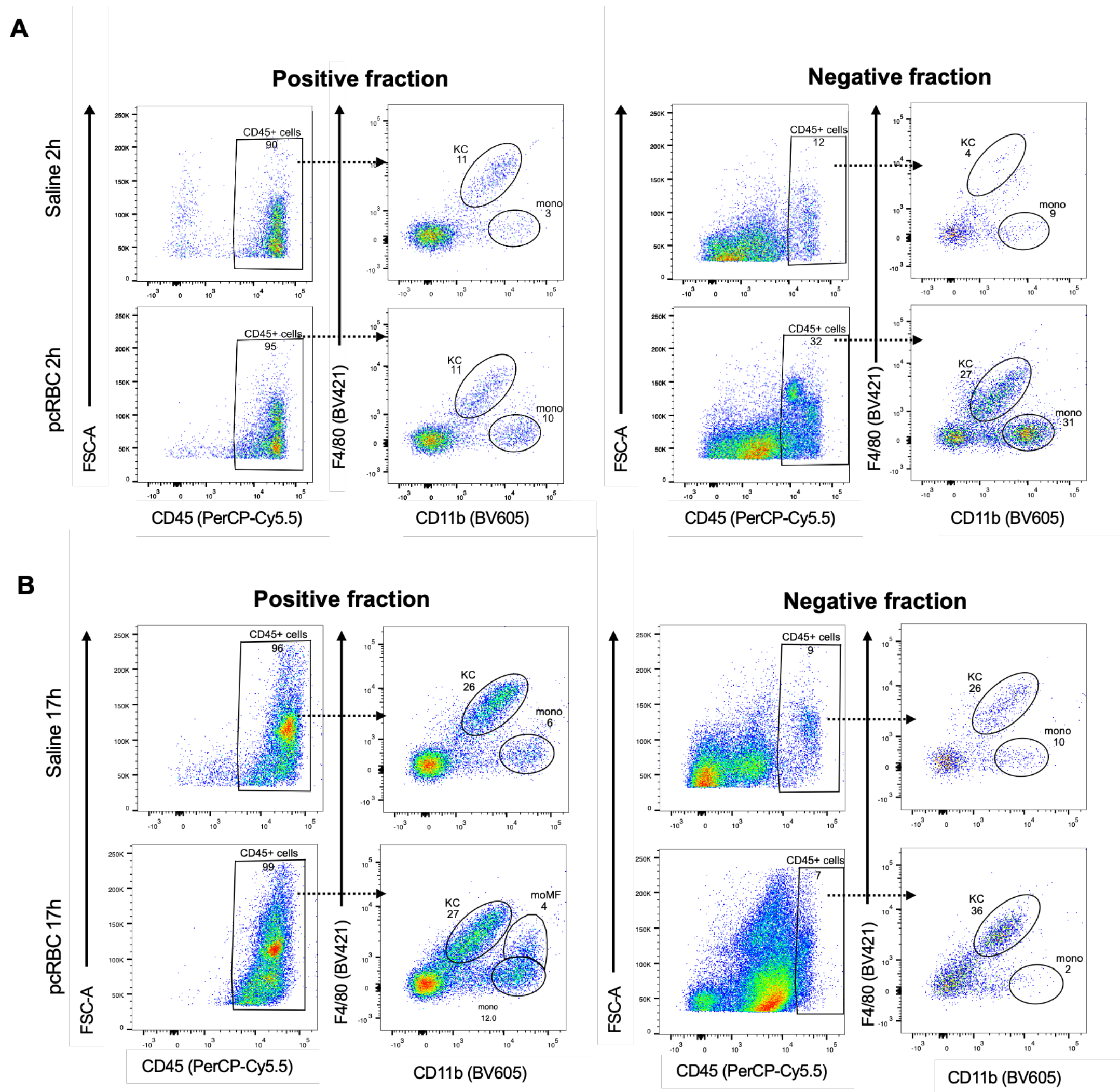

Fig. S5. CD45$^+$ purity control of scRNAseq input data (second run). CD45$^+$ liver cells were processed for scRNAseq as described in Materials and Methods (Cell preparation for scRNAseq analysis). Quality control of the positive (left) and negative (right) fractions of liver NPC post CD45$^+$ enrichment of saline 2 h and pcRBC 2 h samples **(A)** and saline 17 h and pcRBC 17 h samples **(B)**. The cells are gated as FSC-SSC single live CD45$^+$ cells.

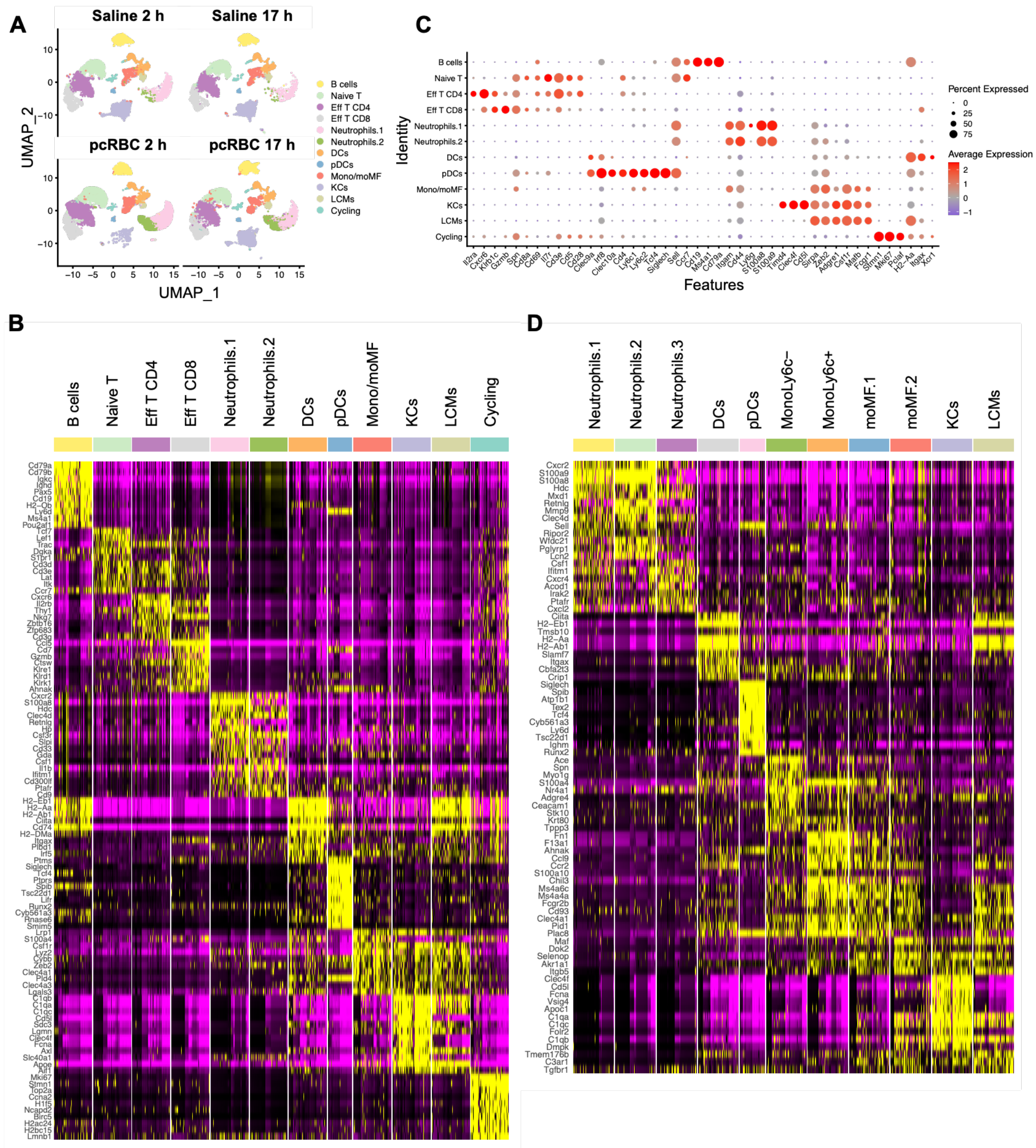

Fig. S6. pcRBC treatment alters the hepatic myeloid cell equilibrium: a single-cell analysis. **(A)** UMAP demonstrating all 4 conditions assessed by scRNAseq analysis. Saline 2 h, saline 17 h, pcRBC 2 h, and pcRBC 17 h are depicted. 12 clusters are portrayed. **(B)** Heatmap depicting the top 10 markers expressed in each identified cluster (12 clusters). **(C)** Dot plot depicting gene expression levels and detection frequency of the cluster-defining genes of all CD45^{+} cells. **(D)** Heatmap depicting the top markers expressed per cluster after re-clustering the dataset only for myeloid cells (10 clusters). Details regarding the clustering can be found in Materials and Methods, under *Integration and Clustering*.

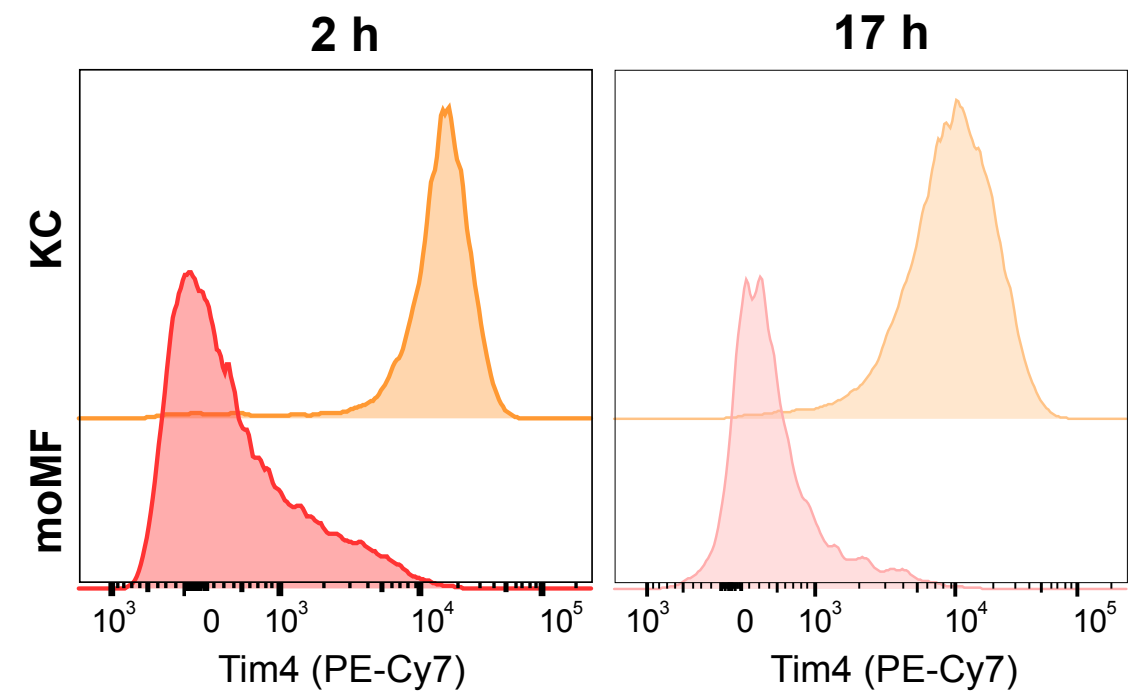

Fig. S7. Increased TIM4 expression in KC compared to infiltrating moMF after pcRBC treatment. Representative histograms of TIM4 expression in KC (saline-treated mouse) and moMF (pcRBC-treated mouse). Gating was performed according to **Fig. S9**. Data representative of 3 independent experiments (n= 8) for the 2 h and 2 independent experiments (n=6) for the 17 h timepoint.

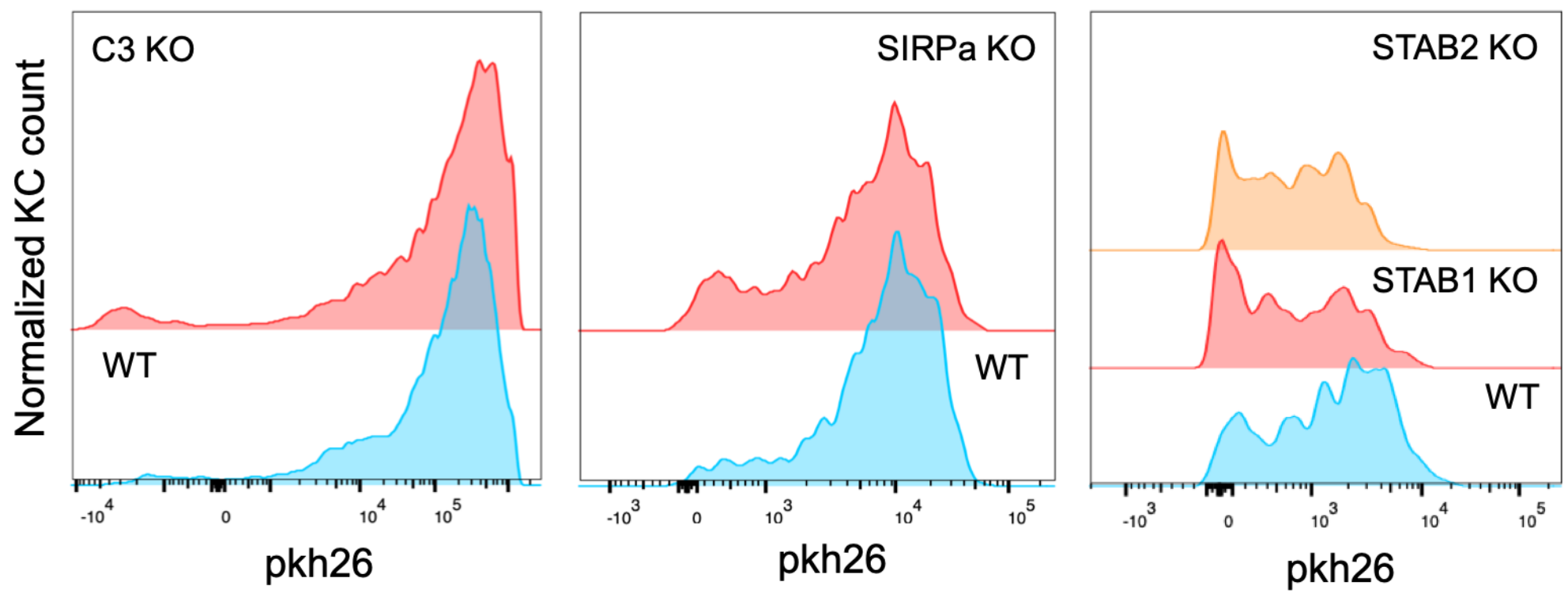

Fig. S8. Mechanisms of pcRBC phagocytosis. Representative histograms depicting the fluorescence intensity of pkh26 in pkh26^{+}CD11b^{+} KC for the different KO mice and their WT littermates of each KO-WT experiment. Data representative of 2-3 independent experiments (n=3-15/group).

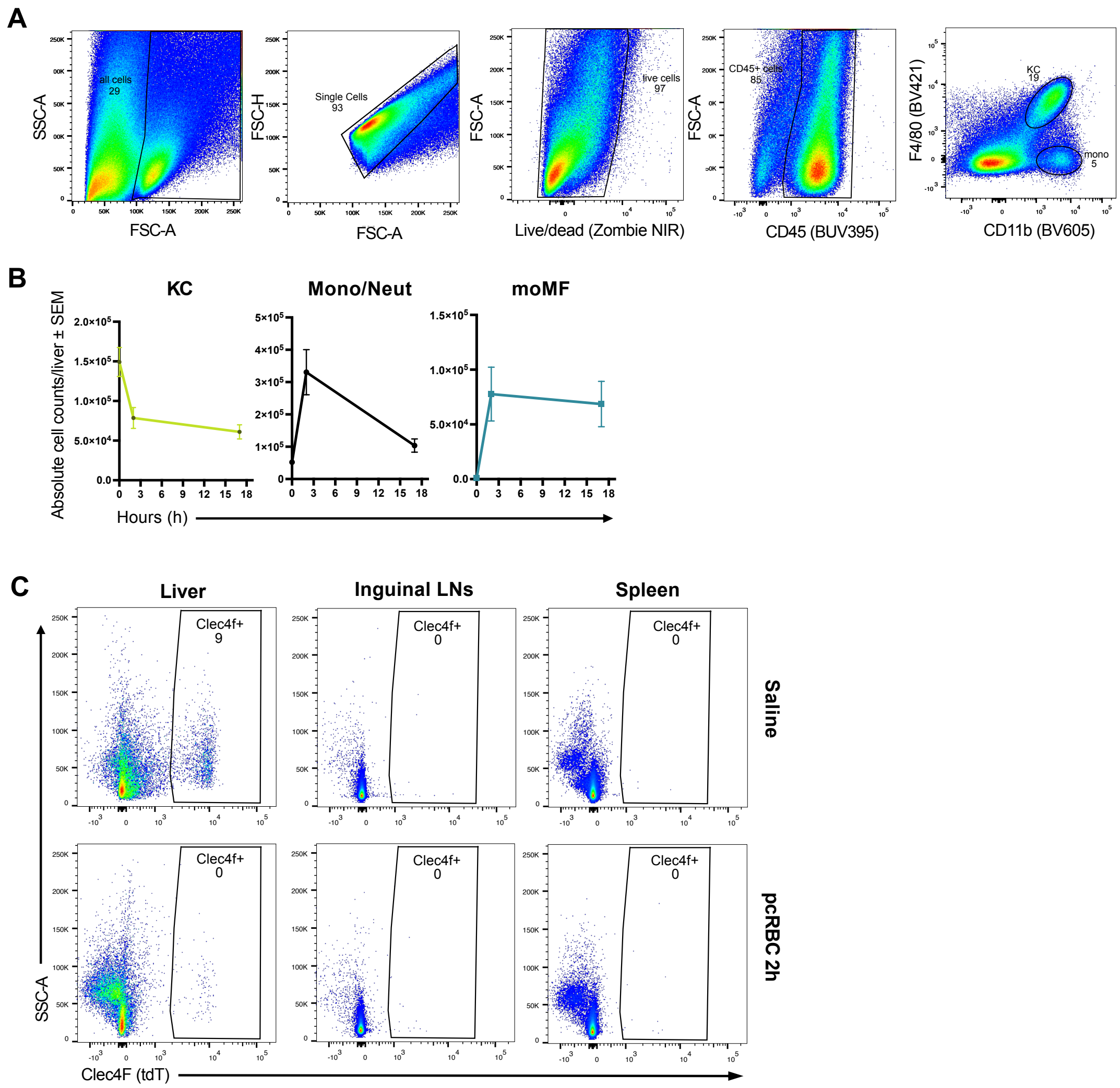

Fig. S9. Liver myeloid cell dynamics post pcRBC application. (A) Gating strategy of liver KC and monocytes at steady state: FSC-SSC gate → single cells → live cells → CD45$^+$ cells. **(B)** XY plots of absolute numbers of KC, Mono/Neut and moMF in saline versus 2 h and 17 h post pcRBC injection. For the timepoint of 0 h, the absolute numbers of the respective populations from the saline condition across all experiments included in this analysis were pooled. The absolute cell counts were calculated as NPC count*gated events/total of recorded events. Data from 4-6 independent experiments (n=12-21/timepoint). **(C)** Representative FACS plots of liver NPC, inguinal LNs, and spleen single-cell suspensions of saline control and pcRBC-treated animals (2 h post-application). Percentages are representative of a normalized number of CD45$^+$ cells. Data representative of 1 experiment, n=2 (saline), n=3 (pcRBC).

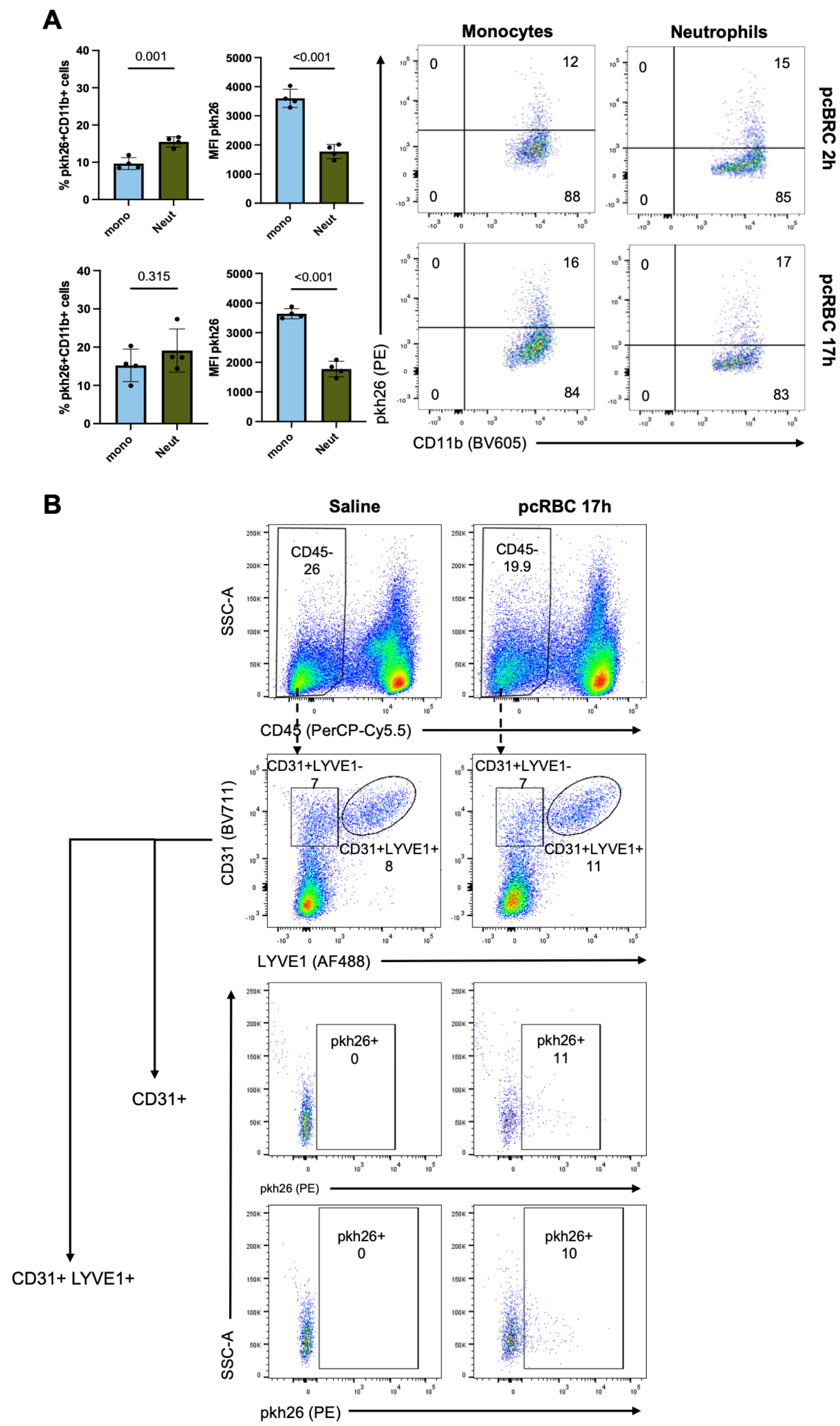

Fig. S10. Assessment of pcRBC uptake by selected liver non-parenchymal cell populations. (A) Uptake of pkh26-labeled pcRBC by monocytes and neutrophils 2 h and 17 h post pcRBC injection. Bar plots of %pkh26$^+$CD11b$^+$ monocytes and neutrophils, MFI pkh26 within the pkh26$^+$CD11b$^+$ populations (mean ± SD), and representative FACS plots. An unpaired t-test was performed to compare the means of the two groups. Statistical significance: $p < 0.05$. **(B)** Uptake of pkh26-labeled pcRBC by LSECs 17 h post injection. Representative FACS plots of pkh26$^+$ CD31$^+$LYVE1$^-$ and CD31$^+$LYVE1$^+$ LSECs. Data from 1 experiment (n=2).

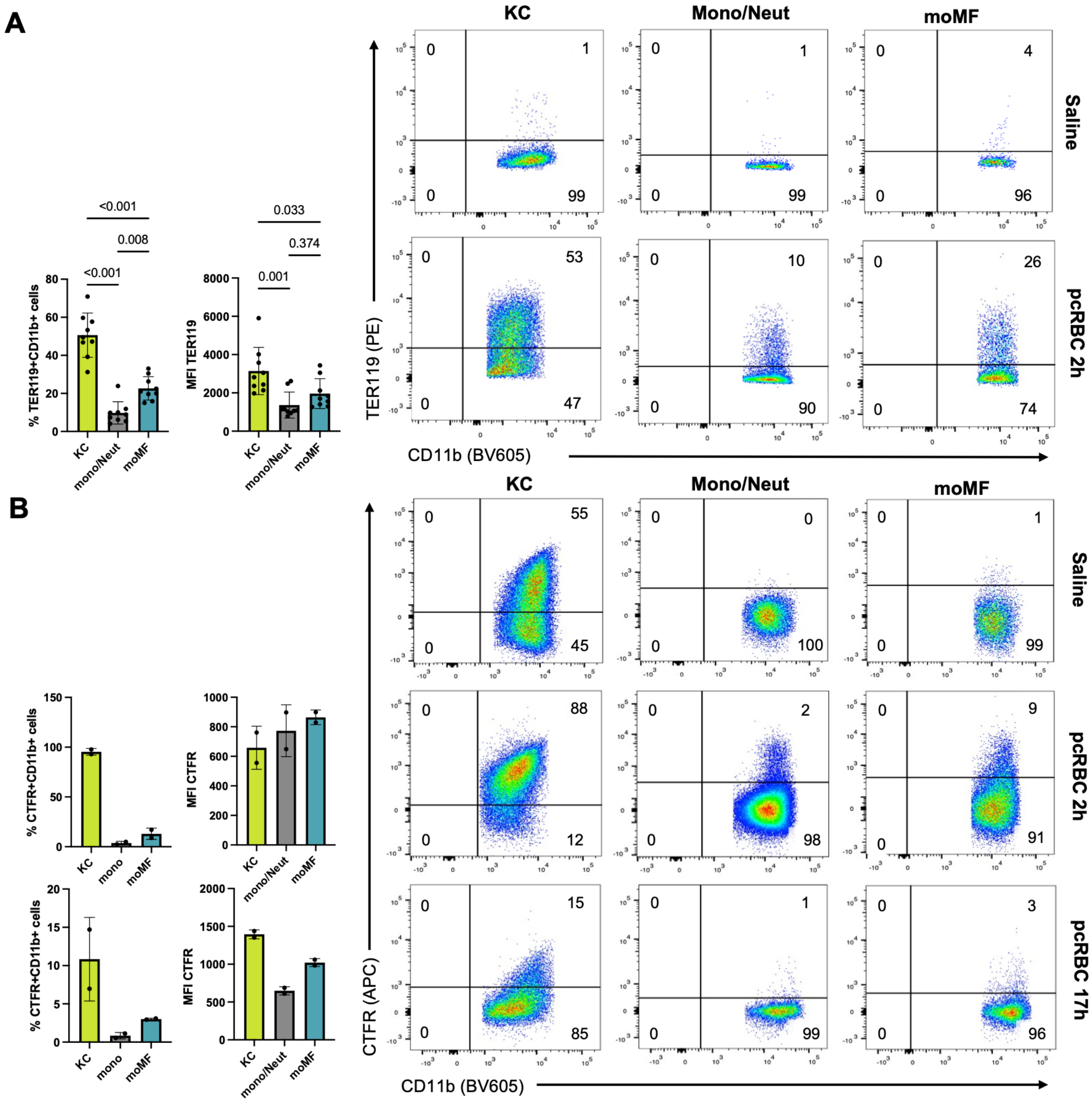

Fig S11. Validation of pcRBC labelling and detection of uptake by liver phagocytes. (A) Uptake of pcRBC by KC, mono, and moMF 2 h post pcRBC injection, assessed by intracellular TER119 staining. Bar plots of %TER119$^+$CD11b$^+$ cells and MFI of TER119 in TER119$^+$CD11b$^+$ populations (mean ± SD) and representative FACS plots. An ordinary one-way ANOVA with multiple comparisons was performed. Statistical significance: $p < 0.05$. **(B)** Uptake of CTFR-labeled pcRBC by KC, mono, and moMF 2 h and 17 h post pcRBC injection. Bar plots of %CTFR$^+$CD11b$^+$ cells and MFI of CTFR in CTFR$^+$CD11b$^+$ populations (mean ± SD) and representative FACS plots.

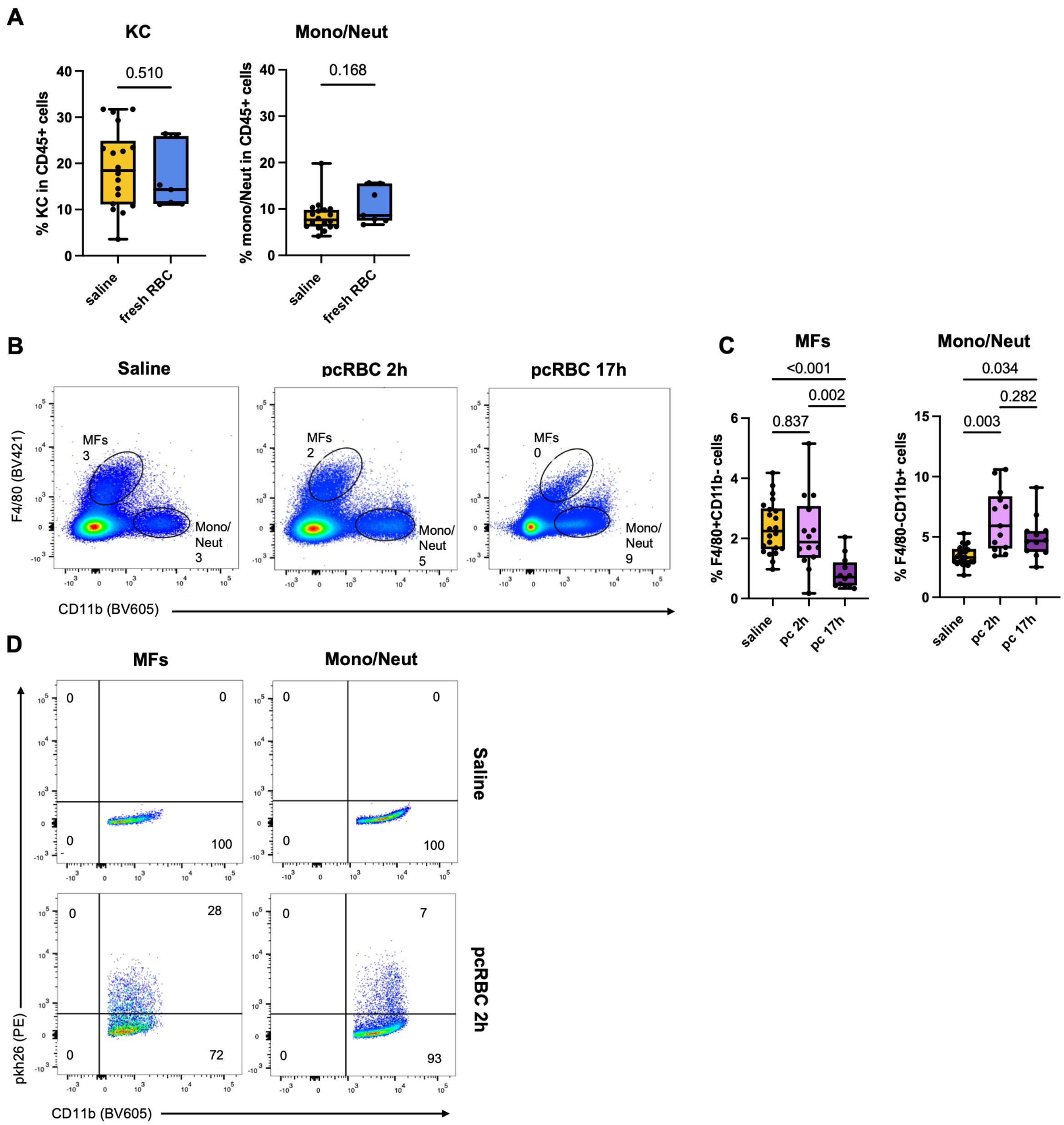

Fig. S12. Uptake of fresh RBC in liver and pcRBC in spleen. (A) Percentage of KC and mono/Neut 2 h after injection of fresh RBC or 0.9% saline (control). An unpaired t-test was performed to compare the 2 groups. **(B)** Representative FACS plots of spleen myeloid populations post saline and 2 h and 17 h post pcRBC injection. **(C)** Percentage of spleen macrophages (MFs) and mono/Neut 2 h and 17 h post injection of pcRBC or 0.9% saline (control). A one-way ANOVA, and Brown-Forsythe and Welch ANOVA tests, with multiple comparisons, were performed for the MFs and Mono/Neut groups, respectively. Statistical significance: $p < 0.05$. **(D)** Representative FACS plots of pkh26$^+$ MFs and Mono/Neut 2 h post pkh26-labeled pcRBC injection.

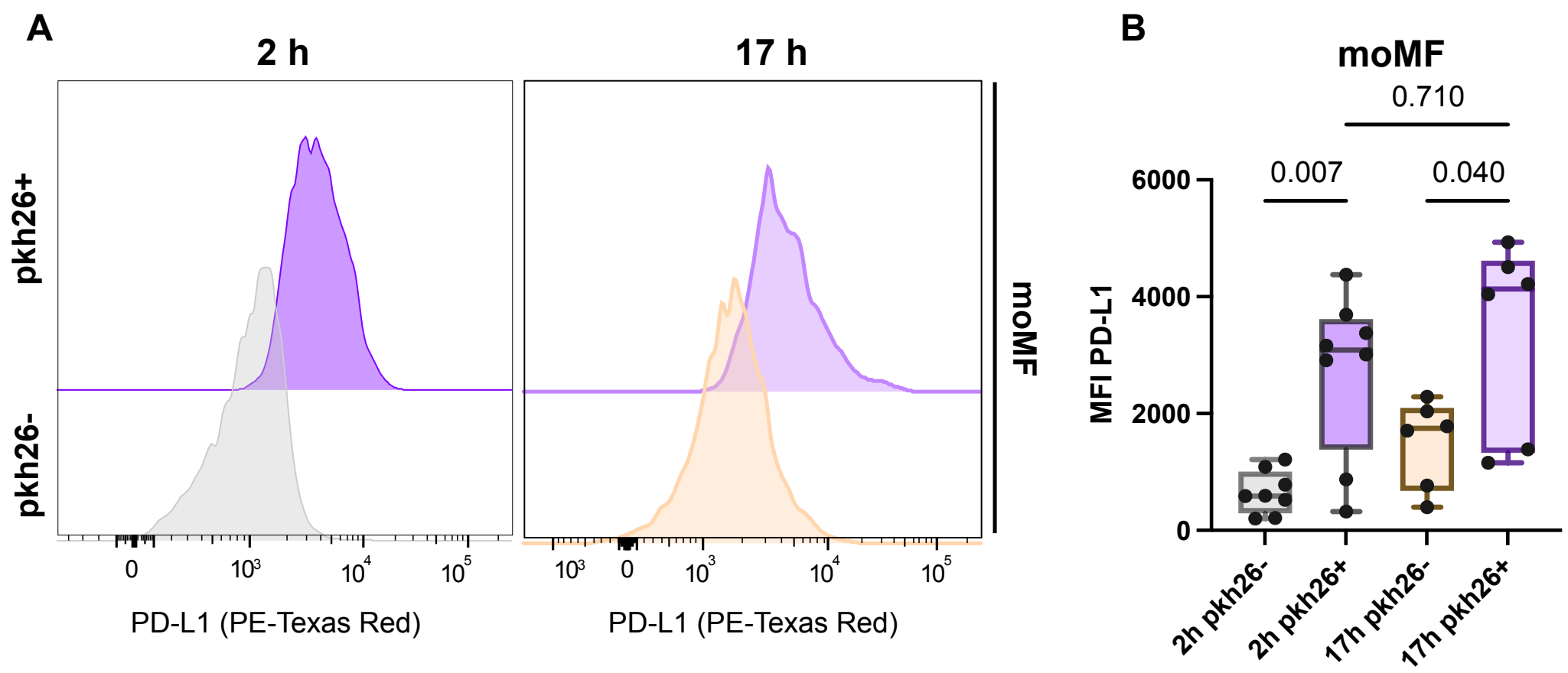

Fig. S13. PD-L1 expression is increased in pcRBC-phagocytosing moMF. (A) Representative histograms of PD-L1 expression in pkh26- and pkh26$^+$ moMF 2 h (left) and 17 h post pcRBC treatment (right). **(B)** PD-L1 mean fluorescence intensity (MFI) in pkh26$^-$ and pkh26$^+$ moMF at the different time points. Data from 3 independent experiments for 2 h (n= 8) and 2 independent experiments for 17 h timepoint (n= 6). Ordinary ANOVA with multiple comparisons was performed. Statistical significance: $p < 0.05$.

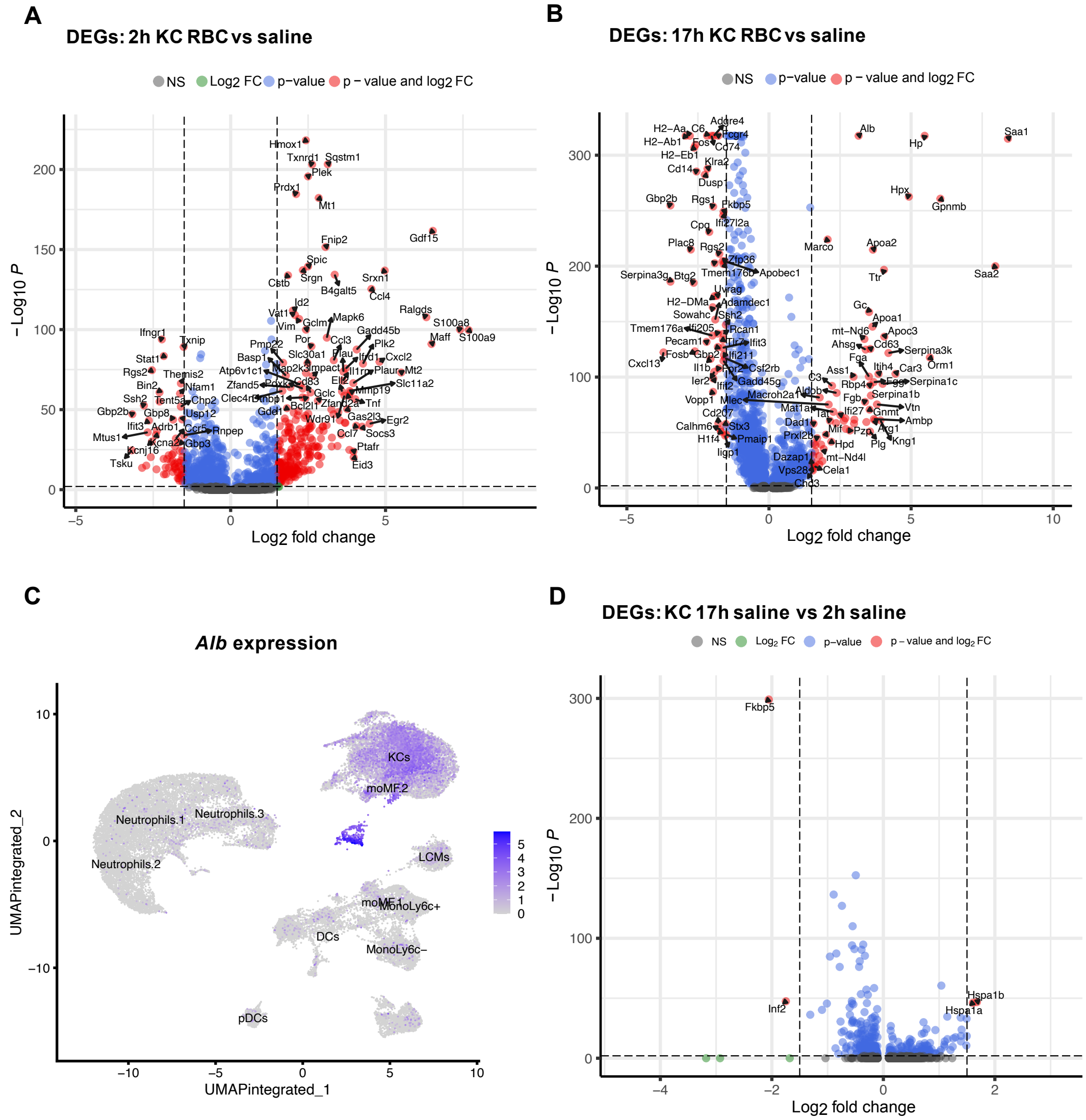

Fig. S14. pcRBC treatment alters the phenotype of liver KC. Differential gene expression (DEG) analysis was performed for KC between different conditions. Volcano plot depicting the DEGs between pcRBC 2 h and saline 2 h **(A)** and pcRBC 17 h and saline 2 h **(B)**. **(C)** UMAP depicting *Alb* expression across pcRBC and saline samples. **(D)** Volcano plot depicting the DEGs between saline 2 h and saline 17 h. DEGs were considered significant and relevant with FDR < 0.01 and |logFC| >= 1.5.

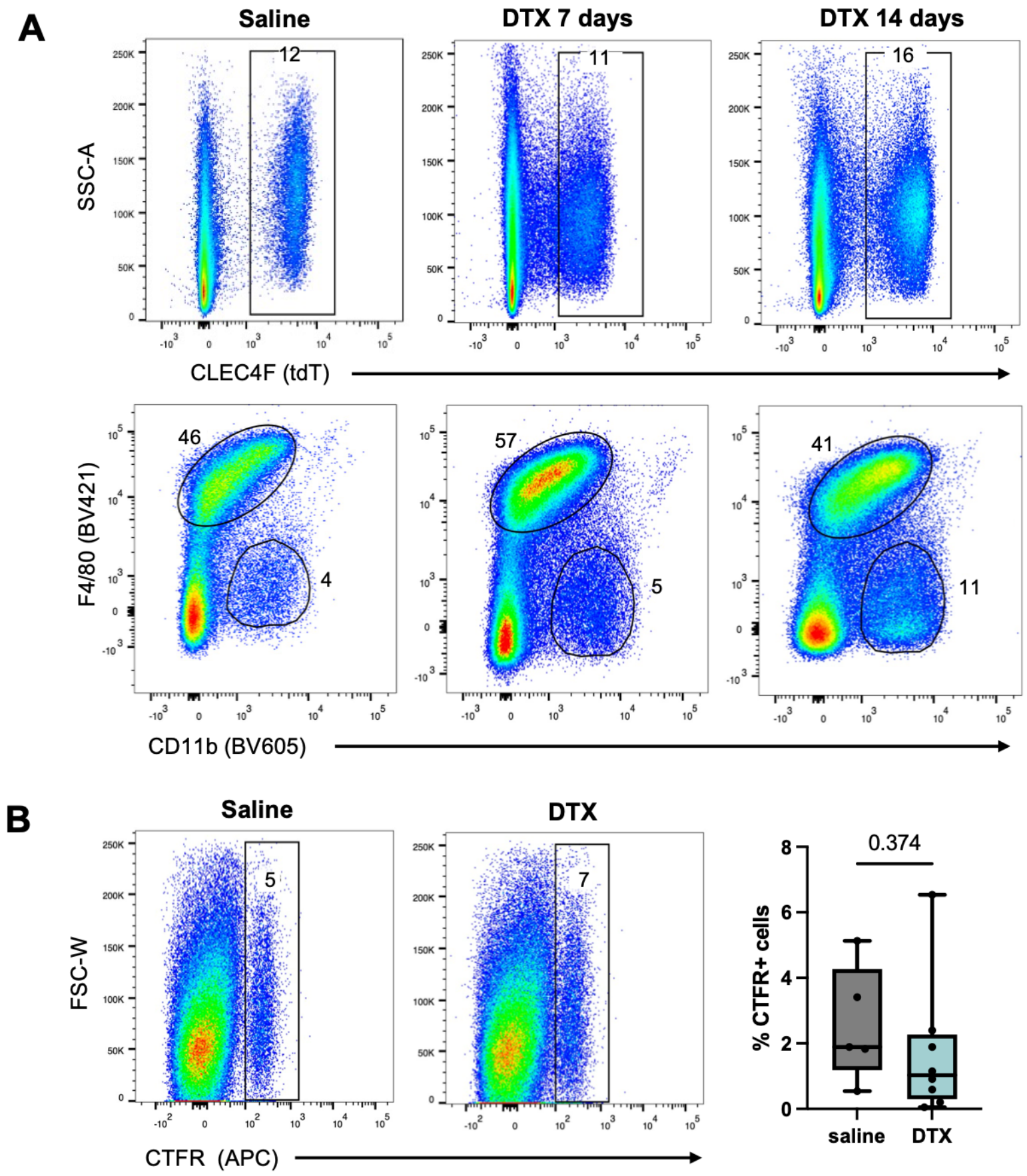

Fig. S15. Kupffer cell depletion in KC-DTR mice does not affect circulating pcRBC levels. KC-DTR mice were i.p. treated with 100 ng DTx and were assessed for the presence of KC. **(A)** Representative FACS plots of KC in KC-DTR mice, assessed by tdTomato signal (CLEC4F) and F4/80 and CD11b staining seven days and fourteen days post DTx treatment. The representative F4/80-CD11b saline plot is identical to that shown in **Fig. 5A** for ease of comparison. **(B)** Representative FACS plots and percentages of $CTFR^{+}$ cells (CTFR-labeled pcRBC) in the blood of DTx- or saline-treated KC-DTR animals. Mann-Whitney non-parametric test was applied for the comparison of the two groups. Statistical significance: $p < 0.05$.

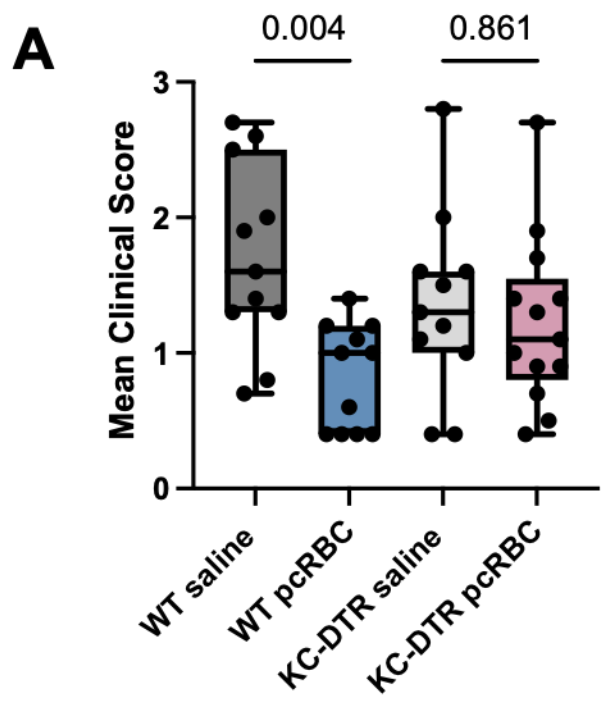

B

Genotype	Treatment	N	Incidence (%)	AUC± SEM	Day Onset (Mean ±STD)	Day Peakdisease (Mean ±STD)	Maxscore (Mean)
WT	saline	11	100	42.4±3.7	7.7±1.3	15,72±1,68	2.9
WT	pcRBC	11	100	20.5±2.1	8±1.3	15,5±5,73	1.7
KC-DTR	saline	11	100	33.9±3.5	8.6±1.6	16,5±2,73	2.6
KC-DTR	pcRBC	13	100	30.6±3.2	9.1±1.6	15,5±1,94	2.4

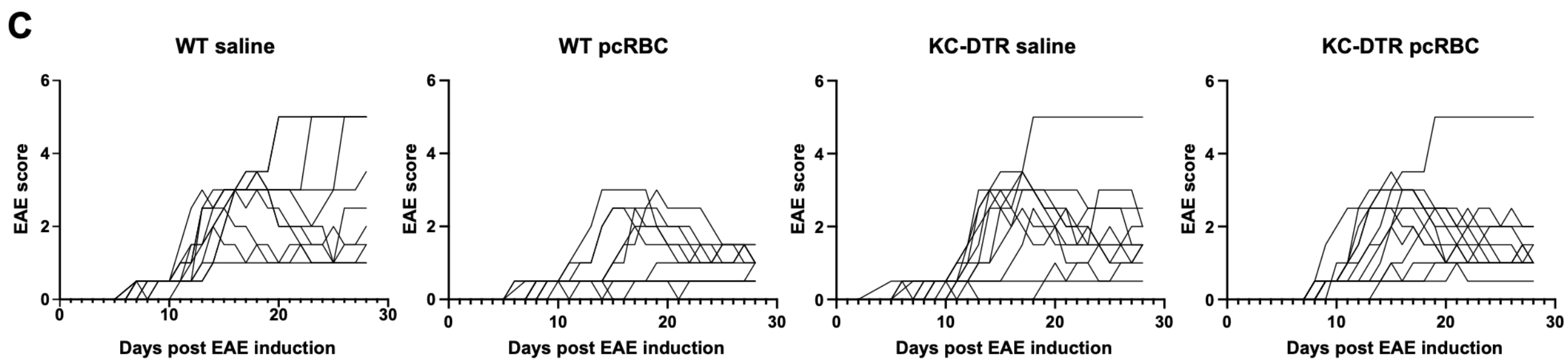

Fig. S16. KC depletion abrogates tolerance induction. (A) Mean clinical scores of the different treatment groups of EAE induction post DTx and pcRBC/saline administration. Brown-Forsythe and Welch ANOVA tests were applied to compare WT saline versus WT pcRBC and KC-DTR saline versus KC-DTR pcRBC groups. Statistical significance: $p < 0.05$. **(B)** Number of mice (N), Area under the curve (AUC), Day of onset, day of peak disease, and highest score per genotype per treatment. **(C)** Spaghetti plots of disease development of individual mice per group.

Table S1.

Name of Line	Common Name	Abbreviation used	Producer/Reference
C57BL/6J	WT	WT	Bred at University of Zurich, Switzerland or purchased from Janvier (Rj) (C57BL-6JRj)
C57BL/6-Clec4f$^{em1(cre)}$Glass/J Gt(ROSA)Sor$^{tm1(HBEGF)Awai}$/J	Clec4f-Cre-iDTR	Clec4f-iDTR	Bred at University of Zurich, Switzerland
B6.129S4-C3tm1Crr/J	C3KO	C3KO	Obtained from SWIMMR list
B6.129P2-Sirpa^{tm1Nog}/Rbrc	SIRPα KO	SIRPα KO	Bred at University of Zurich, Switzerland
B6;129S2-Stab1$^{tm1.1Cger}$	Stabilin 1 KO	Stabilin 1 KO	Bred at Heidelberg University, Germany
B6;129S2-Stab2$^{tm1.1Cger}$	Stabilin 2 KO	Stabilin 2 KO	Bred at Heidelberg University, Germany
C57BL/6-*Mavs*^{em1Thbu}	MAVS KO	MAVS KO	Bred at University of Zurich, Switzerland
C57BL/6-*Sting1*^{em1ThBu}	STING KO	STING KO	Bred at University of Zurich, Switzerland
C57BL/6-*Ticam2*^{em1ThBu}	TRAM KO	TRAM KO	Bred at University of Zurich, Switzerland
B6;129S1-*Tlr3*^{tm1Flv}/J	TLR3KO	TLR3 KO	Bred at University of Zurich, Switzerland
B6.129P2-*Tlr4*^{tm1Aki}	TLR4 KO	TLR4 KO	Bred at University of Zurich, Switzerland

WT and transgenic mouse strains, along with their producer/provider and housing status, used in our study.

Table S2.

**Primary Antibodies**	**Catalog number**	**Dilution**
Rat anti-mouse CLEC4F (370901) mAb	MA5-24113	1:50
Rabbit anti-mouse Iba1/AIF-1 (E4O4W) XP® mAb	17198	1:100
Rat anti-mouse TER119 (TER119) mAb	MA1-70078	1:100
Rabbit anti-mouse/human Cleaved Caspase-3 (Asp175) mAb	9661	1:200
Rat anti-mouse Marco (ED31) mAb	GTX39744	1:200
**Secondary Antibodies**		
Alexa Fluor™ Plus 647 Goat anti-Rat IgG	A48265	1:200
Alexa Fluor™ Plus 647 Goat anti-Rabbit IgG	A32733	1:200
Alexa Fluor™ Plus 555 Goat anti-Rat IgG	A48263	1:100

List of antibodies used for multiplex immunofluorescence on the Comet™ platform.

Table S3. All markers CD45$^+$ cells
Table S4. All markers myeloid cells
Table S5. DEG overlap KC
Table S6. DEGs KC
Table S7. GSEA KC pcRBC 2h vs saline 2h
Table S8. GSEA KC pcRBC 17h vs saline 2h
Table S9. GSEA gene sets
Table S10. DEGs KC 17h pcRBC vs moMF.2 pcRBC
Table S11. GSEA KC vs moMF.2
Table S12. GSEA KC vs moMF.1
Table S13. Source data
